# Resistant starch intake facilitates weight loss in humans by reshaping the gut microbiota

**DOI:** 10.1038/s42255-024-00988-y

**Published:** 2024-02-26

**Authors:** Huating Li, Lei Zhang, Jun Li, Qian Wu, Lingling Qian, Junsheng He, Yueqiong Ni, Petia Kovatcheva-Datchary, Rui Yuan, Shuangbo Liu, Li Shen, Mingliang Zhang, Bin Sheng, Ping Li, Kang Kang, Liang Wu, Qichen Fang, Xiaoxue Long, Xiaolin Wang, Yanli Li, Yaorui Ye, Jianping Ye, Yuqian Bao, Yueliang Zhao, Guowang Xu, Xinyu Liu, Gianni Panagiotou, Aimin Xu, Weiping Jia

**Affiliations:** 1 Shanghai Key Laboratory of Diabetes Mellitus, Department of Endocrinology and Metabolism, Shanghai Diabetes Institute, Shanghai Clinical Center for Diabetes, Shanghai Sixth People’s Hospital Affiliated to Shanghai Jiao Tong University School of Medicine, Shanghai, China; 2 State Key Laboratory of Pharmaceutical Biotechnology, The University of Hong Kong, Hong Kong S.A.R., China; 3 Department of Medicine, The University of Hong Kong, Hong Kong S.A.R., China; 4 Department of Medicine, Shanghai Jiao Tong University School of Medicine, Shanghai, China; 5 Department of Infectious Diseases and Public Health, City University of Hong Kong, Hong Kong S.A.R., China; 6 Department of Microbiome Dynamics, Leibniz Institute for Natural Product Research and Infection Biology - Hans Knöll Institute, Jena, Germany; 7 Cluster of Excellence Balance of the Microverse, Friedrich Schiller University Jena, Jena, Germany; 8 Institute for Molecular Infection Biology, University of Wurzburg, Wurzburg, Germany; 9 College of Food Science and Technology, Shanghai Ocean University, Shanghai, China; 10 Department of Clinical Nutrition, Shanghai Sixth People’s Hospital Affiliated to Shanghai Jiao Tong University School of Medicine, Shanghai, China; 11 Department of Computer Science and Engineering, Shanghai Jiao Tong University, Shanghai, China; 12 Department of Computing, The Hong Kong Polytechnic University, Hong Kong S.A.R., China; 13 School of Design, The Hong Kong Polytechnic University, Hong Kong S.A.R., China; 14 CAS Key Laboratory of Separation Science for Analytical Chemistry, Dalian Institute of Chemical Physics, Chinese Academy of Sciences, Dalian, China; 15 Metabolic Disease Research Center, Zhengzhou University Affiliated Zhengzhou Central Hospital, Zhengzhou, China; 16 School of Public Health, Shanghai Jiao Tong University School of Medicine, Shanghai, China; 17 Faculty of Biological Sciences, Friedrich Schiller University, Jena, Germany; 18 Department of Microbiology, Li Ka Shing Faculty of Medicine, The University of Hong Kong, Hong Kong S.A.R., China

**Keywords:** Obesity, Clinical microbiology, Metabolism, Microbiota, Randomized controlled trials

## Abstract

Emerging evidence suggests that modulation of gut microbiota by dietary fibre may offer solutions for metabolic disorders. In a randomized placebo-controlled crossover design trial (ChiCTR-TTRCC-13003333) in 37 participants with overweight or obesity, we test whether resistant starch (RS) as a dietary supplement influences obesity-related outcomes. Here, we show that RS supplementation for 8 weeks can help to achieve weight loss (mean −2.8 kg) and improve insulin resistance in individuals with excess body weight. The benefits of RS are associated with changes in gut microbiota composition. Supplementation with *Bifidobacterium* *adolescentis*, a species that is markedly associated with the alleviation of obesity in the study participants, protects male mice from diet-induced obesity. Mechanistically, the RS-induced changes in the gut microbiota alter the bile acid profile, reduce inflammation by restoring the intestinal barrier and inhibit lipid absorption. We demonstrate that RS can facilitate weight loss at least partially through *B.* *adolescentis* and that the gut microbiota is essential for the action of RS.

## Abstract (web summary)

In a randomized placebo-controlled trial in 37 individuals with excess body weight, dietary supplementation with resistant starch lowers body weight and induces changes in gut microbiota composition. Mechanistic analysis in male mice shows that resistant starch at least partially facilitates weight loss through the action of *Bifidobacterium* *adolescentis*.

## Main

Owing to the current global obesity epidemic, research is directed towards new obesity prevention and weight-reduction strategies. This is crucial because obesity significantly contributes to comorbidities such as diabetes and cardiovascular diseases—major global mortality causes. Conversely, weight loss mitigates these comorbidities, underscoring weight management’s importance in preventing and treating these conditions^1^.

The gut microbiota has been increasingly recognized as an important regulator of host physiology and pathophysiology^2^. Specifically, previous studies have reported that gut microbiota regulates inflammation, fat storage and glucose metabolism^3,4^. Although the results of faecal microbial transplantation (FMT) from healthy donors to individuals with obesity were inconsistent or short-term^5,6^, combining dietary intervention with FMT could result in favourable alterations in the recipients’ microbiota and improvements in clinical outcomes^7^. Thus, the rational manipulation of the gut microbiome by dietary interventions might be a promising anti-obesity strategy^2,7^.

Prebiotics, including polysaccharides, oligosaccharides and other fermentable dietary fibres, increase the amount of beneficial gut microbiota, notably certain *Bifidobacterium* and *Lactobacillus* spp. These bacteria diminish pathogen populations, fortify the gut barrier and mitigate the inflammatory response^8^. Moreover, snacks formulated with different fibre preparations could be tailored to alter functions associated with specific elements of the microbiome^9^; however, most prebiotic studies were based on correlations without establishing a causal link between the modulation of the gut microbiota and the observed beneficial effects on metabolism^2^. Insights from both human trials and mechanistic studies in gnotobiotic animals are crucial to establish the causality between microbiome alterations and host biological responses. Furthermore, these studies are vital to comprehend the mechanisms connecting microbiome changes to the physiological advantages of prebiotics or other fermentable dietary fibres^2,9,10^.

RS refers to a kind of fermentable dietary fibre that cannot be digested by human amylases in the small intestine and moves into the colon, where it undergoes fermentation by gut microbiota^11^. Studies in rodents have demonstrated RS could lead to a decrease in total body fat, particularly visceral fat, as opposed to digestible starch feeding^12^. Diets low in protein and high in carbohydrate yield the most favourable metabolic outcomes when the carbohydrate component consists of RS in mice^13^; however, human data showed that there was no impact on the total body weight of individuals with metabolic syndrome after being fed RS for a duration spanning 4 to 12 weeks^14–16^. Low-fat diets supplemented with RS had beneficial effects on the hosts, but high-fat diets attenuated the RS fermentation and the beneficial effects^17,18^. This may be one possible explanation as to why RS seemingly had no impact on body weight in the clinical trials described above, as those clinical trials did not have a high compliance rate to the diet^12,19^. Moreover, this implies RS-associated gut microbiota’s vital role in RS’s therapeutic effects; however, RS’s potential as a functional, adaptable food ingredient for obesity treatment in humans and the modulation of metabolic benefits by RS-related gut microbiome alterations remain unclear. Thus, a robust trial in obese individuals is essential to substantiate claims about RS’s impact on diverse physiological aspects in consumers and the required dosage^9,19^. Moreover, multi-omics approaches and gnotobiotic animal models should be used to systematically and mechanistically connect the influence of RS on the gut microbial community and the host’s metabolism^12,19^.

Here we performed a crossover, randomized clinical trial in individuals with excess body weight to investigate the effect of RS as a dietary supplement on obesity and other metabolic phenotypes. The trial is a feeding study providing isoenergetic and balanced background diets. Metagenomics and metabolomics analyses were conducted to assess RS’s impact on gut microbiota composition and function. Additionally, we analysed the influence of RS-modified gut microbiota transferred from selected human donors to antibiotic-treated mice on host adiposity and glucose metabolism, we also explored the mechanisms underlying the metabolic advantages conferred by gut microbiota through RS.

## Results

### RS intervention facilitates weight loss

**Fig. 1:**
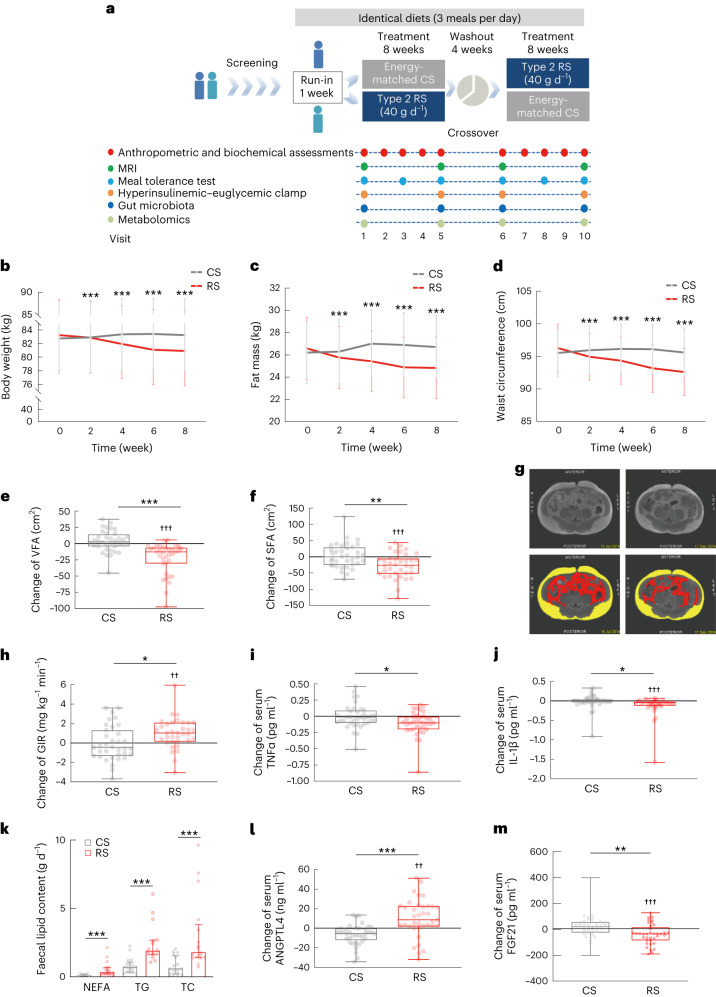
Alleviation of obesity after the 8-week RS intervention in individuals with excess body weight. **a**, Diagram of the clinical trial. After enrolment, randomization and run-in period, participants consumed either RS or CS alternately and separated by a washout period. During the whole trial, all participants were provided with identical diets. The assessments at each visit are displayed in the diagram. **b–d**, RS intervention significantly reduced body weight (**b**), fat mass (**c**) and waist circumference (**d**). **e**,**f**, Change of VFA and SFA evaluated by MRI. **g**, Representative abdominal MRI of participants before (left) and after (right) the 8-week RS intervention. Raw (top) and marked (bottom) MRI at navel level. Yellow represents SFA and red represents VFA. **h**, Change of GIR evaluated by hyperinsulinemic–euglycemic clamp. **i**, Change of serum TNFα levels. **j**, Change of serum IL-1β levels. **k**, Daily faecal lipid excretion, including NEFA, TG and TC after the 8-week interventions with RS or CS. **l**, Change of serum ANGPTL4 levels. **m**, Change of serum FGF21 levels. *n* = 37 individuals (**b**–**d**,**j**,**l**,**m**), *n* = 36 individuals (**e**,**f**,**i**), *n* = 35 individuals (**h**) and *n* = 17 individuals (**k**) for either RS or CS. Analysis of covariance (ANCOVA) adjusted by baseline value was used for comparison between RS and CS at each visit (**b**–**d**). Data are shown as mean (95% confidence interval (CI)). ***P < 0.001. Data are shown as median with IQR (**k**). Nonparametric Wilcoxon rank-sum test was used to evaluate the significance between the two interventions. ***P < 0.001. Data are shown as box-and-whisker plots (**e**,**f**,**h**–**j**,**l**,**m**). Box plot, median and quartiles; whiskers, data range. *P = 0.025, 0.014 and 0.046 (**h**–**j**), **P = 0.004 and 0.002 (**f**,**m**), ***P < 0.001 for the between-group difference assessed by the linear mixed model adjusted for intervention order. ^††^*P* = 0.003 and 0.002 (**h**,**l**). ^†††^*P* < 0.001 for the within-group change by mixed linear model adjusted for intervention followed by Bonferroni’s test.

**Extended Data Fig. 1:**
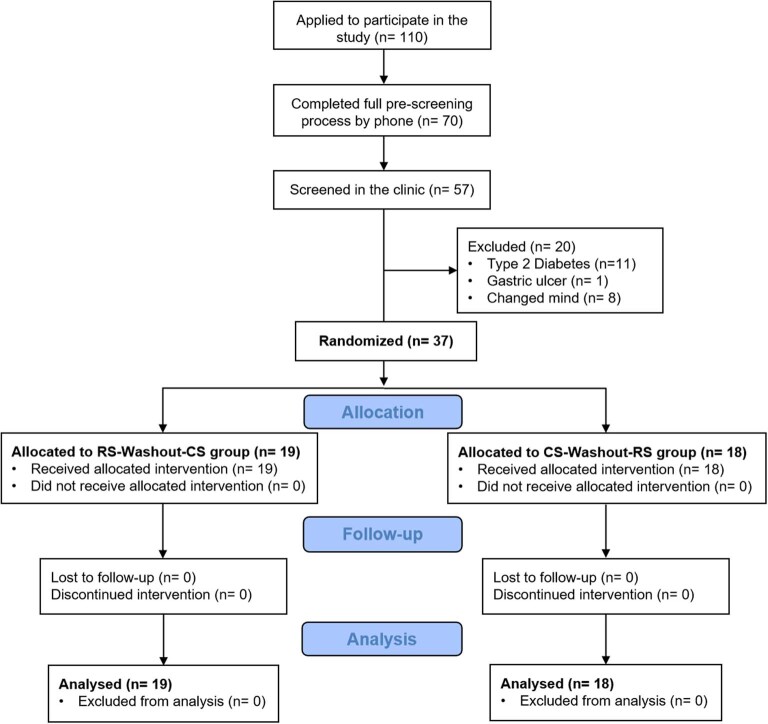
CONSORT diagram. CONSORT flow diagram illustrating the procedures for enrolment, intervention allocation, follow-up and data analysis of the study. RS, resistant starch; CS, control starch.

The investigation was a placebo-controlled, double-blinded, crossover design intervention (ChiCTR-TTRCC-13003333), involving 37 participants (average age of 33.43 ± 7.71 years). The participants had a body mass index (BMI) ≥ 24 kg m^−2^ and/or increased waist circumference (≥85 cm for men and ≥80 cm for women). None had chronic disorders, ongoing treatments impacting glucose metabolism or recent use of antibiotics or probiotic (within 3 weeks). The 20-week study duration included two intervention periods of 8 weeks, with one each for high-amylose maize (HAM-RS2) (RS, 2.8 kcal g^−1^, 91.2 g, containing 40 g RS) and control starch (CS) (AMIOCA) (3.55 kcal g^−1^, 72 g, amylopectin, containing 0 g RS, with equal energy supply) and a 4-week washout period between the interventions (Fig. 1a). Study participants were randomly allocated into two groups: (1) RS-Washout-CS or (2) CS-Washout-RS. Starch was provided as powder in pre-packaged sachets to be mixed with 300 ml water. Each participants consumed one sachet twice a day, 10–15 min before meals. Throughout the trial, encompassing the run-in, two interventions and the intervening washout period, we provided an isoenergetic and balanced background diet (three meals per day), according to the Chinese and American guidelines for prevention and management of adults with overweight and obesity (details in the study protocol of Supplementary Information)^20,21^. Participants showed no difference in skipping meals or starch consumption between RS and CS interventions, suggesting that there was no difference in dietary compliance between the RS and CS intervention. Except for differences in dietary fibre intake (RS versus CS, 53.84 ± 4.70 g versus 9.97 ± 5.17 g, *P* < 0.001), total energy consumed and percentage of macronutrients were similar during the RS or CS intervention periods (Supplementary Table 1). In total, 37 participants, including 22 male and 15 female participants finished the study and were incorporated into the analyses (Table 1 and Extended Data Fig. 1). The study was conducted in Shanghai, China from 3 July 2013 to 14 October 2016 and reported no gastrointestinal side effects, such as nausea, vomiting, bloating, increased bowel movement or change of stool frequency.

**Table 1:** Summary of clinical parameters of the study participants before and after CS or RS intervention

Clinical parameters	CS 0 W	CS 8 W	RS 0 W	RS 8 W	*P* value^a^	*P* value^b^	*P* value^c^	*P* value^d^
**Obesity-related indicators**								
Body weight (kg)	82.74 ± 15.67	83.23 ± 16.01	83.24 ± 15.70	80.91 ± 15.39	0.328	0.339	<0.001	<0.001
BMI (kg m^−2^)	28.58 ± 3.79	28.74 ± 3.95	28.75 ± 3.79	27.93 ± 3.75	0.361	0.422	<0.001	<0.001
FM (kg)	26.21 ± 8.37	26.71 ± 8.45	26.59 ± 8.34	24.83 ± 8.29	0.486	0.136	<0.001	<0.001
FFM (kg)	56.54 ± 12.33	56.57 ± 12.46	56.66 ± 12.35	55.75 ± 11.99	0.999	0.999	0.011	0.029
TBW (kg)	39.55 ± 7.71	39.51 ± 7.82	39.61 ± 7.69	39.10 ± 7.68	0.999	0.999	0.101	0.155
Fat percentage	31.70 ± 7.72	31.80 ± 8.03	31.99 ± 7.64	31.06 ± 7.93	0.999	0.999	0.101	0.117
Waist circumference (cm)	95.52 ± 10.91	95.59 ± 11.16	96.28 ± 11.00	92.60 ± 10.89	0.731	0.999	<0.001	<0.001
Hip circumference (cm)	105.00 ± 8.05	105.49 ± 7.71	105.31 ± 8.08	104.16 ± 7.69	0.999	0.372	<0.001	<0.001
Waist-to-hip ratio	0.91 ± 0.06	0.91 ± 0.07	0.91 ± 0.06	0.89 ± 0.06	0.999	0.999	<0.001	0.01
VFA (cm²)	104.75 ± 53.46	108.31 ± 49.71	108.58 ± 55.06	86.39 ± 45.41	0.999	0.999	<0.001	<0.001
SFA (cm²)	283.63 ± 92.87	286.68 ± 96.69	289.64 ± 95.38	262.50 ± 89.91	0.999	0.999	<0.001	0.004
**Anthropometric parameters**								
Age (years)	33.43 ± 7.71							
Sex (male:female)	22:15							
SBP (mm Hg)	112.65 ± 10.00	113.51 ± 8.70	112.41 ± 11.71	113.05 ± 11.19	0.999	0.999	0.999	0.918
DBP (mm Hg)	76.14 ± 7.08	77.11 ± 6.88	75.08 ± 7.69	74.46 ± 7.54	0.999	0.999	0.999	0.342
**Liver enzymes and renal function**								
ALT (U l^−1^)	16.80 (12.30, 29.00)	20.00 (10.28, 30.25)	16.00 (12.00, 30.00)	15.00 (12.00, 22.50)	0.999	0.999	0.432	0.041
AST (U l^−1^)	18.60 (17.20, 22.00)	19.76 (16.03, 24.80)	19.00 (17.00, 22.50)	17.00 (14.50, 21.00)	0.999	0.999	0.058	0.022
GGT (U l^−1^)	24.80 (17.50, 35.00)	25.20 (18.17, 34.26)	25.00 (18.00, 36.00)	21.00 (16.00, 35.50)	0.999	0.999	0.033	0.03
BUN (mmol l^−1^)	4.40 ± 0.97	4.54 ± 1.17	4.38 ± 0.99	4.23 ± 1.06	0.999	0.999	0.999	0.149
Cr (μmol l^−1^)	70.52 ± 12.94	70.26 ± 16.11	70.62 ± 13.02	69.95 ± 14.00	0.999	0.999	0.999	0.809
**Lipid profiles**								
TC (mmol l^−1^)	4.65 ± 0.67	4.56 ± 0.80	4.69 ± 0.67	4.40 ± 0.71	0.999	0.999	0.008	0.146
TG (mmol l^−1^)	1.09 (0.86, 1.68)	1.23 (0.80, 1.52)	1.04 (0.85, 1.64)	1.01 (0.79, 1.38)	0.999	0.999	0.999	0.559
HDL-C (mmol l^−1^)	1.14 ± 0.24	1.14 ± 0.23	1.13 ± 0.25	1.10 ± 0.20	0.999	0.999	0.999	0.456
LDL-C (mmol l^−1^)	3.01 ± 0.58	2.95 ± 0.67	3.02 ± 0.60	2.89 ± 0.54	0.999	0.999	0.424	0.482
**Secretory cytokines**								
A-FABP (ng ml^−1^)	35.49 (30.71, 44.82)	36.83 (23.35, 46.99)	37.47 (29.33, 47.81)	31.12 (23.74, 45.12)	0.999	0.999	0.072	0.12
Adiponectin (μg ml^−1^)	9.34 (7.49, 13.86)	9.38 (7.01, 12.39)	9.21 (7.04, 13.13)	10.46 (7.38, 13.68)	0.999	0.999	0.376	0.038
FGF21 (pg ml^−1^)	167.61 (99.71, 211.00)	172.07 (104.63, 237.70)	188.46 (113.19, 224.07)	151.74 (51.62, 216.98)	0.999	0.999	<0.001	0.002
**Inflammation-related factors**								
TNFα (pg ml^−1^)	0.70 (0.60, 0.81)	0.68 (0.55, 0.86)	0.70 (0.55, 0.82)	0.66 (0.45, 0.72)	0.999	0.999	0.053	0.014
IL-1β (pg ml^−1^)	0.18 (0.13, 0.24)	0.17 (0.10, 0.27)	0.16 (0.11, 0.26)	0.11 (0.06, 0.17)	0.999	0.999	<0.001	0.046
MCP-1 (pg ml^−1^)	673.56 (535.55, 865.83)	630.62 (516.20, 768.22)	626.85 (513.23, 816.09)	631.97 (498.41, 822.20)	0.999	0.999	0.999	0.925
IL-10 (pg ml^−1^)	1.72 (1.58, 2.07)	1.62 (1.49, 1.79)	1.66 (1.52, 1.95)	1.58 (1.48, 1.91)	0.999	0.999	0.999	0.146
IL-6 (pg ml^−1^)	1.08 (0.65,1.85)	1.12 (0.57,1.64)	1.08 (0.77,1.58)	1.05 (0.71,1.49)	0.999	0.999	0.999	0.689

FM, fat mass; FFM, free fat mass; TBW, total body water; SBP, systolic blood pressure; DBP, diastolic blood pressure; ALT, alanine transaminase; AST, aspartate transaminase; GGT, γ-glutamyl transferase; HDL-C, high-density lipoprotein cholesterol; LDL-C, low-density lipoprotein cholesterol; BUN, blood urea nitrogen; Cr, creatinine; A-FABP; adipocyte fatty acid-binding protein. Data are expressed as mean ± s.d. or median (interquartile range (IQR)).

^a^Differences in baseline variables before the RS and CS interventions were assessed using a linear mixed model adjusted by intervention order followed by Bonferroni’s test.

^b^Differences in outcomes before and after CS intervention were assessed using a linear mixed model adjusted by intervention order followed by Bonferroni’s test.

^c^Differences in outcomes before and after RS intervention were assessed using a linear mixed model adjusted by intervention order followed by Bonferroni’s test.

^d^Differences in outcomes between the RS and CS intervention were assessed using a linear mixed model adjusted by intervention order.

**Extended Data Fig. 2:**
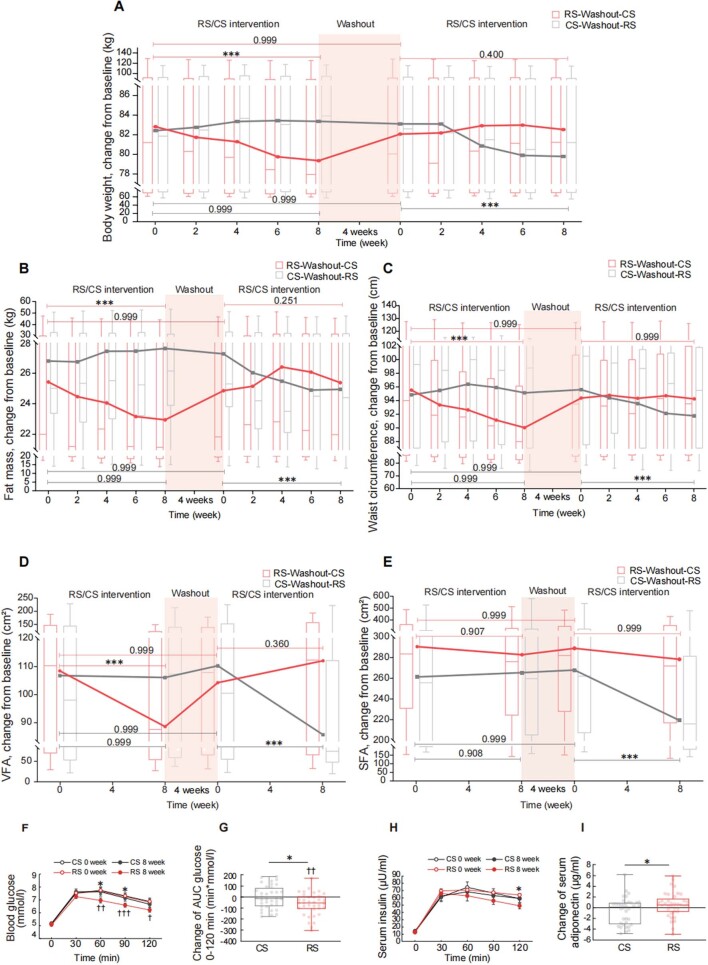
Improvement of obesity and glucose metabolism after the 8-week RS intervention in individuals with excess body weight. (**a-e**) Boxplots (with median) showing the dynamic changes of (**a**) body weight, (**b**) fat mass, and (**c**) waist circumference at every 2 weeks of intervention, as well as (**d**) visceral fat (VFA) and (**e**) subcutaneous fat area (SFA) at every 4 weeks of intervention. Lines connect the means of outcomes at different time pointes. The horizontal line represents the outcomes of the linear mixed model analysis followed by Bonferroni’s test conducted to compare the connected endpoints. RS-Washout-CS group, n = 19 individuals. CS-Washout-RS group, n = 18 individuals. *** *P* < 0.001. (**f-h**) Standardized meal tolerance tests (MTT) were performed at baseline (0 week) and after 8-week RS or CS intervention. (**f**) Blood glucose. (**g**) Change of area under curve (AUC) and analysis of blood glucose. (**h**) Serum insulin. (**i**) Change of serum adiponectin level evaluated by ELISA in subjects before and after 8-week RS or CS intervention. For **f**-**h**, n = 36 individuals per group, and for **i**, n = 37 individuals per group. Data are expressed as mean ± s.e.m (**f**, **h**). Data are shown as box-and-whisker plots (**g**, **i**). The horizontal line in the middle of each box indicates the median value, the top and bottom borders of the boxes represent the 75th and 25th percentiles, and the whiskers denote the lowest and highest values. * *P* = 0.023 and 0.020 (**f**), 0.016 (**g**), 0.036 (**h**), and 0.038 (**i**), for the difference between RS and CS intervention by linear mixed model adjusted by intervention order and † *P* = 0.022, †† *P* = 0.001 (**f**) and 0.001 (**g**), ††† *P* < 0.001 (**f**) for the difference before and after RS intervention (RS 0W vs. RS 8W) by linear mixed model adjusted by intervention order followed by Bonferroni’s test.

We compared the actual changes in anthropometric parameters and biochemical indices between the interventions (RS versus CS) and the within-group change before and after the intervention using the linear mixed model adjusted by intervention order (Table 1 and Supplementary Table 2). The primary outcome body weight was significantly decreased after the RS intervention and the net absolute change after RS intervention relative to CS intervention was −2.81 kg (95% CI −3.55 kg to −2.07 kg; *P* < 0.001), whereas no significant change was observed after the CS intervention (Fig. 1b). Moreover, fat mass and waist circumference reduced significantly after the RS intervention compared with the CS intervention (Fig. 1c,d). During the RS intervention period, the body weight, waist circumference and fat mass of participants significantly decreased from week 2 onwards. Both visceral fat areas (VFA) and subcutaneous fat areas (SFA), measured by abdominal magnetic resonance imaging (MRI) were lower following the RS consumption compared with those following CS consumption (*P* < 0.001 and *P* = 0.004, respectively; Fig. 1e–g). Regardless of whether it was in the RS-Washout-CS group or the CS-Washout-RS group, significant reductions were observed in body weight and other obesity-related outcomes after the RS intervention (all *P* < 0.05). In the RS-Washout-CS group, obesity-related outcomes showed a reversion to baseline levels after the washout period (Supplementary Table 3 and Extended Data Fig. 2a–e). Moreover, the two-way analysis of variance (ANOVA) showed significant difference in body weight and obesity-related outcomes (fat mass, waist circumference and VFA) on intervention (*P* < 0.001) and no significant order effect or intervention-order interaction. These results demonstrated that an 8-week RS intervention reduced abdominal adiposity in individuals with excess body weight.

Furthermore, glucose tolerance improved significantly after the RS intervention (Extended Data Fig. 2f,g). The insulin concentrations at 120 min following a meal tolerance test (MTT) in participants after the RS intervention were significantly lower than those after the CS intervention (Extended Data Fig. 2h); however, the CS intervention did not induce any differences in glucose and insulin levels compared with baseline values (Extended Data Fig. 2f,h). We further assessed insulin sensitivity by hyperinsulinemic–euglycemic clamp and found that the glucose infusion rate (GIR) was significantly increased after the RS intervention (with a median increase of 1.05 mg kg^−1^ min^−1^, 0.15 to 2.10) compared to the CS intervention (*P* = 0.025) (Fig. 1h), demonstrating a significant improvement in insulin sensitivity. Furthermore, a significant increase in serum adiponectin levels was observed after the RS intervention (Extended Data Fig. 2i). In addition, after RS intervention, neither the first-phase nor the second-phase insulin secretion was significantly different from those after CS intervention (Supplementary Table 4). All these results indicated that the 8-week RS intervention improved glucose tolerance and insulin sensitivity in individuals with excess body weight.

To investigate the potential mechanism by which RS facilitates weight loss, we determined the changes in chronic, low-grade inflammatory response and intestinal lipid digestion, which are closely related to obesity^22,23^. Levels of pro-inflammatory cytokines such as serum tumour necrosis factor (TNF)α and interleukin (IL)-1β were found significantly lower in the study participants after RS consumption compared with CS consumption (*P* = 0.014 and *P* = 0.046, respectively), although no significant difference in monocyte chemoattractant protein-1 (MCP-1), IL-10 and IL-6 were observed during RS and CS consumption (Fig. 1i,j and Table 1). Furthermore, we measured faecal lipids of the study participants and found that the daily excretion of faecal non-esterified fatty acid (NEFA), triglycerides (TGs) and total cholesterol (TC) were significantly higher following RS consumption compared with CS consumption (Fig. 1k). As there was no significant difference in fat intake during the RS and CS consumption (Supplementary Table 1), these results suggested that the RS intervention may decrease lipid absorption from the diet. Circulating levels of angiopoietin-like 4 (ANGPTL4), a potential connection between gut and lipid metabolism^3^, were significantly increased in the study participants after the RS consumption compared with CS consumption (Fig. 1l). Serum fibroblast growth factor 21 (FGF21), which was reported to increase in obese status, significantly reduced after RS consumption (Fig. 1m)^24^.

### RS intervention reshapes gut microbiota

**Fig. 2:**
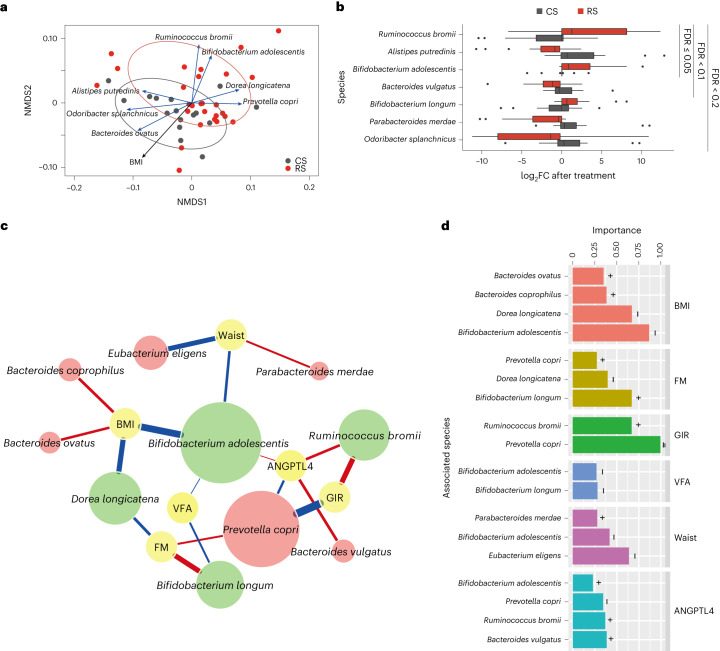
The association of the gut microbiome in response to RS treatment with host phenotypes. **a**, NMDS of the samples based on the variation (fold change after intervention) profile of the species abundance. The red and grey circles surround (with 95% CI) RS and CS samples, respectively. The species and phenotypes correlated with the overall ordination significantly (FDR adjusted *P* < 0.01 with the envfit function in VEGAN R package) are highlighted with arrows (blue, species; black, phenotype) where the length of the arrows reflects the strength of the association. **b**, Seven species with significantly different variation profiles (*P* < 0.05, BH FDR < 0.2 with paired two-sided Wilcoxon signed-rank test) between the RS and CS interventions. Box-plots indicate the median and IQR. Whiskers extend to 1.5 × IQR. **c**, The association network between gut microbes and the host phenotypes. A total of 12 species with different variations (*P* < 0.05, BH FDR < 0.3 with two-sided Wilcoxon rank-sum test) between the RS and CS groups were used as predictors in the regression model. Node colour reflects either the phenotype (yellow), species with increased abundance (green) or species with decreased abundance (red) in response to RS. The colour of the connecting line reflects either the positive correlation (red) or negative correlation (blue) between microbes and the phenotypes. The width of the connecting line reflects the strength of the statistical linear correlation (model averaged importance) between the abundance variation (fold change after intervention) and the variation of the phenotypes (fold change after intervention). The importance is the summed AIC weights of the generalized linear models containing such variables during model selection. The node size of the species reflects its overall influence on all the phenotypes. **d**, Summary of the importance of the associated species for each phenotype. In **a**–**d**, 27 individuals before and after RS and 16 individuals before and after CS were randomly selected from all participants for metagenomic analysis. Source data

**Extended Data Fig. 3:**
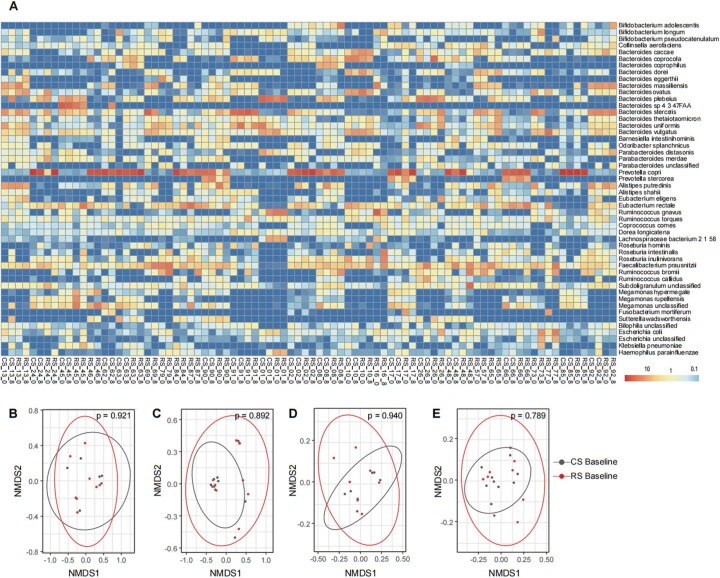
Taxonomic profile and baseline composition of the gut microbiome. (**a**) The taxonomic profile of the gut microbiome at the species level for all the samples from RS and CS intervention. The samples were named using the rules of “Intervention (RS or CS) individual ID_time point (week 0 or 8). (**b-e**) Analysis of baseline microbial composition between RS intervention baseline and CS intervention baseline. (**b**) Beta-diversity for the CS-Washout-RS group using the Bray–Curtis distance method. (**c**) Beta-diversity for the RS-Washout-CS group using the Bray–Curtis distance method. (**d**) Beta-diversity for the CS-Washout-RS group using the weighted-Unifrac distance method. (**e**) Beta-diversity for the RS-Washout-CS group using the weighted-Unifrac distance method. All the beta-diversity plots used Non-metric multidimensional scaling (NMDS) based on the compositional profile of the species abundance. The ANOSIM results support the baseline comparability of microbial compositions (*P* > 0.05). Source data

To investigate the dynamics of the gut microbiota during the interventions, a shotgun metagenomic sequencing was conducted. The average throughput for each sample was 5.74 (s.d. 0.87) Gbp. Species-level taxonomic profiles based on the top 50 species by mean abundance across samples revealed that *Prevotella* *copri*, *Bacteroides* *stercoris* and *Faecalibacterium* *prausnitzii*, were the most prevalent across samples. The other species were present at a much lower abundance (on average 1.1% per species) for the majority of the samples (Extended Data Fig. 3a). To evaluate the extent to which the composition of the gut microbiome was altered in response to the RS intervention, we calculated the Bray–Curtis distance based on the profile of species variation (as fold change in abundance after the 8-week intervention) between samples using the following procedures. The profile of species variation was composed of log_2_ fold change (log_2_ FC) in abundance after treatment for each species. Fold change values were normalized ranging from 0 to 1 with the sum as one in each sample. We further calculated the Bray–Curtis distance based on this profile of species variation. As shown in the non-metric multidimensional scaling (NMDS) plot based on the Bray–Curtis distance, RS and CS samples were separated to a certain extent, indicating significantly different variation patterns in the microbiota after the RS and CS interventions, respectively (*P* < 0.001 with the Adonis test from VEGAN) (Fig. 2a). This finding thus suggests that the RS intervention influences the dynamics and restructures the composition of the gut microbiota. There was no significant difference in microbial composition between the baseline of CS and RS intervention (Extended Data Fig. 3b–e). We found that seven species were significantly correlated (Benjamini–Hochberg (BH) false discovery rate (FDR) adjusted *P* < 0.01) with the overall variation profiles (for all RS and CS samples) (Fig. 2a). We found that BMI significantly correlated (*P* < 0.001 with the envfit test in R package VEGAN) with the overall taxonomic variation profiles (Fig. 2a), suggesting that the change in body weight is associated with the restructured gut microbiome.

To identify the gut microbiota signature associated with the RS intervention, we compared the changes in the abundance of each species after the RS and CS treatments (as a fold change in the abundance after the 8-week intervention). The results showed that three species, *Bifidobacterium* *adolescentis*, *Bifidobacterium* *longum* and *Ruminococcus* *bromii*, were significantly increased after the RS intervention but remained stable or decreased after the CS intervention. Conversely, four species, including *Alisipes* *putredinis*, *Bacteroides* *vulgatus*, *Odoribacter* *splanchnicus* and *Parabacteroides* *merdae* were decreased (*P* < 0.05, BH FDR < 0.2 with a Wilcoxon signed-rank test) after the RS treatment but remained stable or increased after the CS treatment. Among the above, *R.* *bromii, A.* *putredinis* and *B.* *adolescentis* were the most significantly varied species after the RS intervention compared with the CS intervention (FDR = 0.05) (Fig. 2b and Supplementary Table 5). Other degrading species were not significantly varied after the RS intervention in this study (Supplementary Table 6)^25,26^.

**Extended Data Fig. 4:**
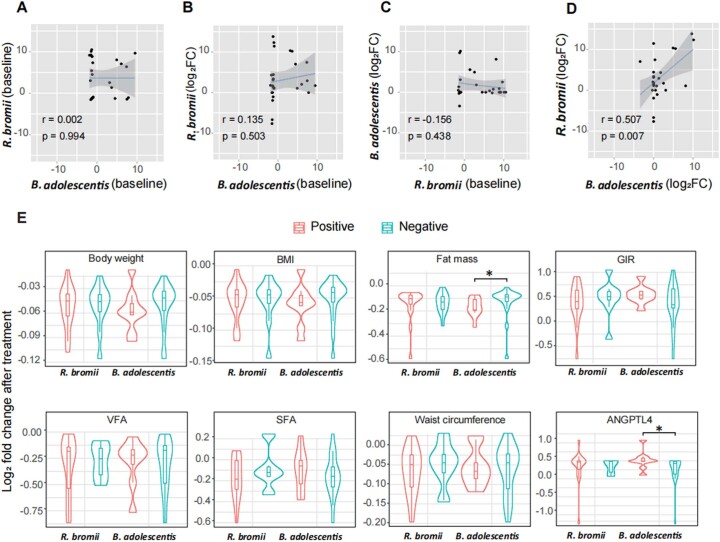
Baseline abundances of two primary degrading microbiota of RS and their relationship with clinical outcomes. (**a-d**) The two-tailed Pearson correlation between (**a**) the baseline abundances of *B. adolescentis* and *R. bromii*. (**b**) the baseline abundances of *B. adolescentis* and the abundance change of *R. bromii*. (**c**) the baseline abundances of *R. bromii* and the abundance change of *B. adolescentis*. (**d**) the abundance changes of *B. adolescentis* and *R. bromii*. The changes in *B. adolescentis* and *R. bromii* are calculated by the log_2_ fold change after treatment. Baseline abundance values have undergone centred log-ratio (CLR) transformation before correlation analysis. The lines and grey zones show the fitted linear regression lines with 95% confidence intervals. (**e**) The relationship between the presence of key species (*B. adolescentis* and *R. bromii*) and the change of key outcomes after RS treatment. The changes of key outcomes were presented by the log_2_ fold change after treatment. The violin plots illustrate the kernel density estimation of the data distribution, and the embedded box plots indicate the median and interquartile range. Whiskers extend to 1.5 times the IQR. n = 16 for *R. bromii* positive individuals, n = 11 for *R. bromii* negative individuals, n = 9 for *B. adolescentis* positive individuals, and n = 18 for *B. adolescentis* negative individuals. Statistical differences between the positive and negative groups were assessed using the two-sided Wilcoxon rank-sum test, with *P* values displayed above the plots. * *P* = 0.033 and 0.046 for fat mass and ANGPTL4 (**e**). The colour indicates the presence (positive, orange) or absence (negative, cyan) of key species. Source data

**Extended Data Fig. 5:**
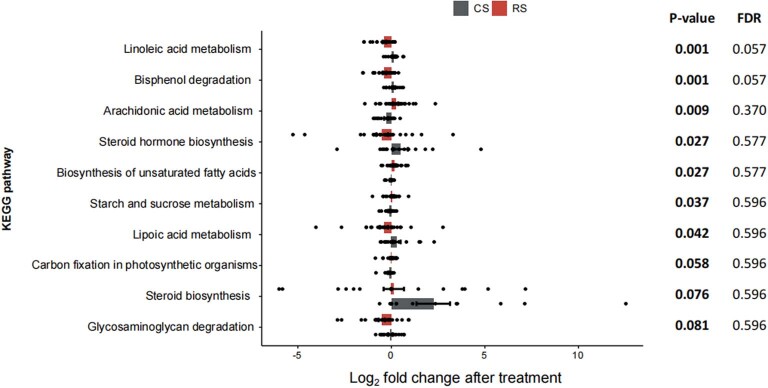
Top 10 metabolism-related KEGG pathways between RS and CS intervention. Data are presented by the log_2_ fold change after treatment, which are expressed as mean ± s.e.m (27 individuals before and after RS and 16 individuals before and after CS). Statistical differences between interventions were assessed using two-sided Wilcoxon rank-sum test, with *P* adjusted for multiple comparisons using the Benjamini–Hochberg method. Source data

To identify species associated with RS intervention benefits, we investigated the correlation of 12 species (FDR < 0.3 via Wilcoxon rank-sum test) with six significantly altered parameters of the host, including BMI, fat mass, waist circumference, VFA, GIR and serum ANGPTL4 levels. A generalized linear model quantified the association between variation of species abundance and variation of the phenotypes. The strength of the association between the species and phenotypes was described by the importance of the variables in the linear model based on the model selection using corrected Akaike information criterion (AIC), which quantified the relative quality/weight of the statistical model used for given data. The importance of a variable reflects the overall support of all possible weighted linear models or the proportion of the total weights for all possible linear models containing this variable. Among the 12 species with differential abundance, *B.* *adolescentis* had the most frequent associations with the various phenotypes (total number of links with the phenotypes) (Fig. 2c). Among the phenotypes, BMI and GIR had the strongest association (average importance > 0.5 for the associated species) with the gut microbes (Fig. 2d). At the single species level, we found that the increased abundance of *R.* *bromii* after supplementation of RS was associated with an increase in GIR (importance 0.67 with *P* < 0.001). Combined with the aforementioned significant increase in *R.* *bromii*, our findings suggested that *R.* *bromii* could be a key species in responding to the RS intervention, consistent with a previous study^27^. *B.* *adolescentis* may play a crucial role in alleviating obesity, evident by its strong correlation with lower BMI, waist circumference and VFA (importance 0.87, 0.47 and 0.27, respectively; *P* < 0.001). Its abundance increases also positively correlated higher serum ANGPTL4 levels (importance 0.252, *P* < 0.001), suggesting potential lipid metabolism effect. As primary RS degraders, the presence of *R.* *bromii* and *B.* *adolescentis* at baseline and their relationship with the key outcomes were explored. The fat mass of *B.* *adolescentis*-positive individuals decreased even more and their ANGPTL4 levels increased even more after RS intervention (both *P* < 0.05), demonstrating that the initial composition of gut microbiota, especially *B.* *adolescentis*, was closely correlated with the benefits of RS (Extended Data Fig. 4). Among the top ten metabolism-related Kyoto Encyclopaedia of Genes and Genomes (KEGG) pathways with the most differently varied abundances between the RS and CS intervention, the linoleic acid metabolism (ko00591) and bisphenol degradation (ko00363) pathways decreased during the RS treatment, whereas they increased significantly in the CS treatment (FDR = 0.057 for both pathways) (Extended Data Fig. 5).

### Association of RS-altered gut microbiota with metabolites

**Extended Data Fig. 6:**
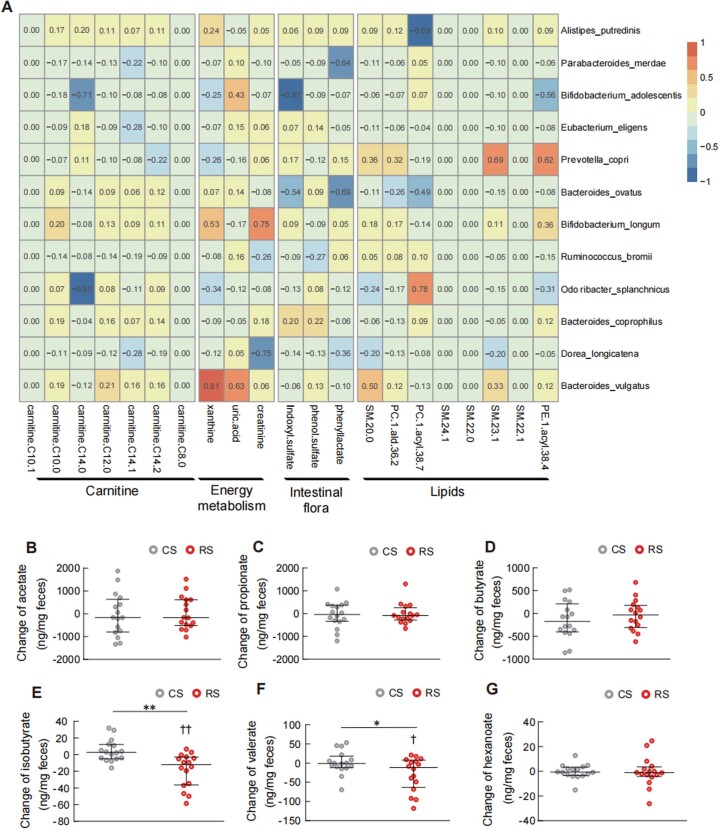
Host metabolomics in response to RS treatment. (**a**) The strength of the linear correlation (model averaged Akaike information criterion [AlC] importance) between the species abundance variation (fold change after intervention) and the variation (fold change after intervention) of the circulating metabolites. The importance is the summed AlC weights of the linear models containing such variable during model selection. (**b-g**) Changes of faecal SCFAs after the 8-week RS or CS intervention (n = 16 individuals per group). (**b**) Acetate. (**c**) Propionate. (**d**) Butyrate. (**e**) Isobutyrate. (**f**) Valerate. (**g**) Hexanoate. Data are expressed as median with interquartile range. * *P* = 0.030 (**f**), ** *P* = 0.006 (**e**) for the difference between RS and CS intervention by linear mixed model adjusted by intervention order and † *P* = 0.047 (**f**), †† *P* = 0.004 (**e**) for the difference before and after RS intervention (RS 0W vs. RS 8W) by linear mixed model adjusted by intervention order followed by Bonferroni’s test. Source data

While it remains unclear how changes in the gut microbiota contribute to benefits in the host, a possible mechanism is through altered metabolic production^28^. Thus, we further performed non-targeted metabolomics profiling in the serum of study participants. Serum metabolites related to obesity, such as carnitine and methionine^29,30^, were decreased after the RS intervention compared with CS intervention (Supplementary Table 7). We used a generalized linear model to investigate the relationship between gut microbes and serum metabolite variation during the RS intervention. The shift of *B.* *adolescentis* during the RS intervention negatively correlated with the shift of serum carnitine, energy metabolism and lipid metabolites (average importance 0.15; Fisher’s combined *P* < 0.05; Extended Data Fig. 6a). In contrast, the shift of *B.* *vulgatus* during the RS intervention positively correlated with the shift of metabolites from energy metabolism, including xanthine and uric acid (*P* < 0.01; Extended Data Fig. 6a).

**Fig. 3:**
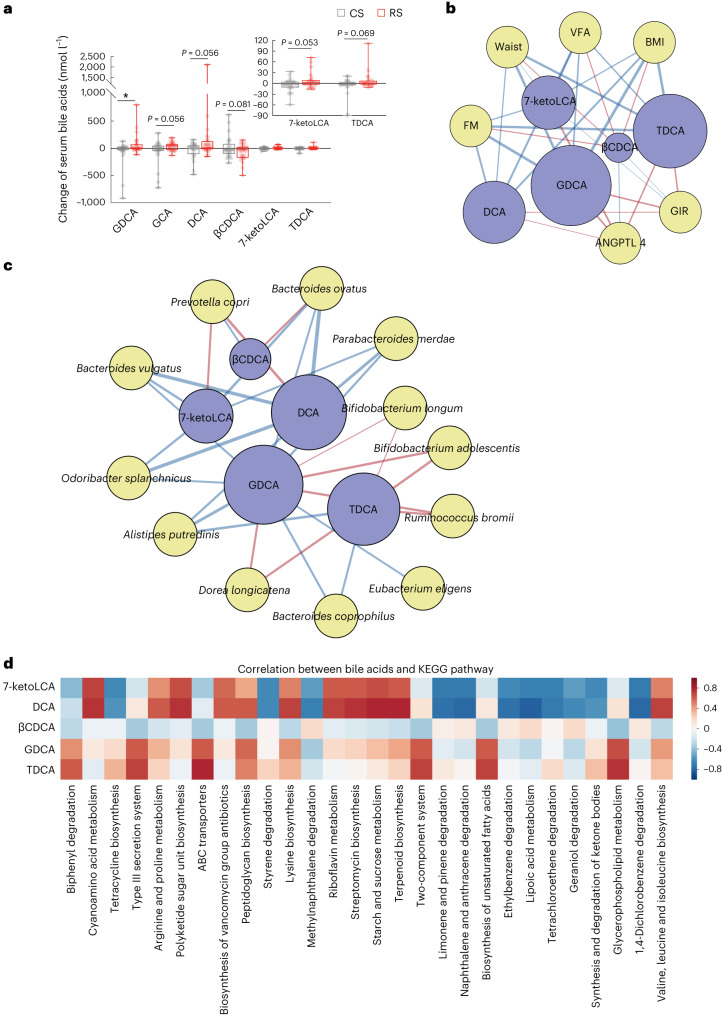
Bile acids mediate the interplay between the gut microbiome and host phenotypes. **a**, Fold change of serum bile acid levels evaluated in participants after an 8-week RS or CS intervention. Data are presented as box-and-whisker plots. The horizontal line in the middle of each box indicates the median value, the top and bottom borders of the boxes represent the 75th and 25th percentiles and the whiskers denote the lowest and highest values. *P = 0.036 by mixed linear model. **b**, The association network between bile acids and the host phenotypes. **c**, The association network between gut microbes and the host phenotypes, respectively. Node colour reflects either the bile acids (blue), the phenotype (yellow in **b**), or the microbes (yellow in **c**). The colour of the connecting line reflects either the positive correlation (red) or negative correlation (blue) between bile acids and phenotypes or between bile acids and microbes. The width of connecting line reflects the strength of the statistical linear association (model averaged importance) between the variation of bile acids (fold change after intervention) and the variation of the phenotypes (fold change after intervention) or between the abundance variation (fold change after intervention) and the variation of the bile acids (fold change after intervention). The importance is the summed AIC weights of the generalized linear models containing such variables during model selection. The node size of the bile acid reflects its overall influence on all the phenotypes or the microbes. **d**, KEGG pathway analysis showing the correlation between the variation of bile acids and the variation of KEGG pathway abundance. Source data

As bile acids are significant signalling metabolites linking gut microbiota with the host, we conducted quantitative analysis of bile acid profiles. Glycodesoxycholic acid (GDCA) was the only bile acid with a statistically significant difference between the RS and CS interventions. Other bile acids, such as glycocholic acid (GCA), deoxycholic acid (DCA), 7-ketolithocholic acid (7-ketoLCA) and taurodeoxycholic acid (TDCA) exhibited a tendency to increase, whereas 3β-chenodeoxycholic acid (βCDCA) level tended to decrease after the RS intervention (Supplementary Table 8 and Fig. 3a). Through correlation analysis, it was discerned that the secondary bile acids GDCA and TDCA had the closest correlation with metabolic phenotypes. The change of GDCA and TDCA was negatively correlated with obesity-related phenotypes, whereas positively correlated with insulin sensitivity and ANGPTL4 levels (Fig. 3b). The change of GDCA and TDCA after RS treatment was significantly and positively associated with four species, including *B.* *adolescentis*, *B.* *longum*, *Dorea* *longicatena* and *R.* *bromii*, whereas negatively associated with another eight species, including *A.* *putredinis*, *Bacteroides* *coprophilus*, *Bacteroides* *ovatus*, *B.* *vulgatus*, *Eubacterium* *eligens*, *O.* *splanchnicus*, *P.* *merdae* and *P.* *copri* (Fig. 3c). Additionally, we identified some microbial pathways strongly associated with the variation of several metabolites, including GDCA, TDCA, βCDCA, DCA and 7-ketoLCA (Fig. 3d). Our results suggest that RS-influenced gut microbiota increases the production of secondary bile acids, which might lead to alleviation of obesity and insulin resistance. To determine whether the species were associated with bile acid metabolism, we investigated the association between gut microbiome and bile salt hydrolase (BSH; K01442), which is the gatekeeper of bile acid metabolism and host–microbiome crosstalk^31^. Our results revealed a significant reduction in the abundance of the *BSH* gene within the gut microbial community level in the RS intervention compared with the CS intervention (*P* < 0.05, Wilcoxon rank-sum test). We found that the expression of *BSH* was significantly correlated with *B.* *vulgatus* (*r* = 0.561, *P* < 0.001; Supplementary Table 9).

Short-chain fatty acids (SCFAs), primarily produced by gut microbial fermentation of dietary fibre, play an important role in the maintenance of gut and metabolic health^28^. Therefore, we conducted targeted metabolomics to investigate the changes in faecal and circulating SCFAs after the RS and CS treatments. We found that faecal concentrations of isobutyrate and valerate significantly decreased after the RS intervention compared with the CS intervention, whereas other faecal SCFAs, including acetate, propionate, butyrate and hexanoate did not differ significantly after the RS intervention (Extended Data Fig. 6b–g). There was no significant difference in circulating levels of SCFAs between the RS and CS interventions (Supplementary Table 10).

### RS-reshaped gut microbiota alleviates obesity in mice

**Fig. 4:**
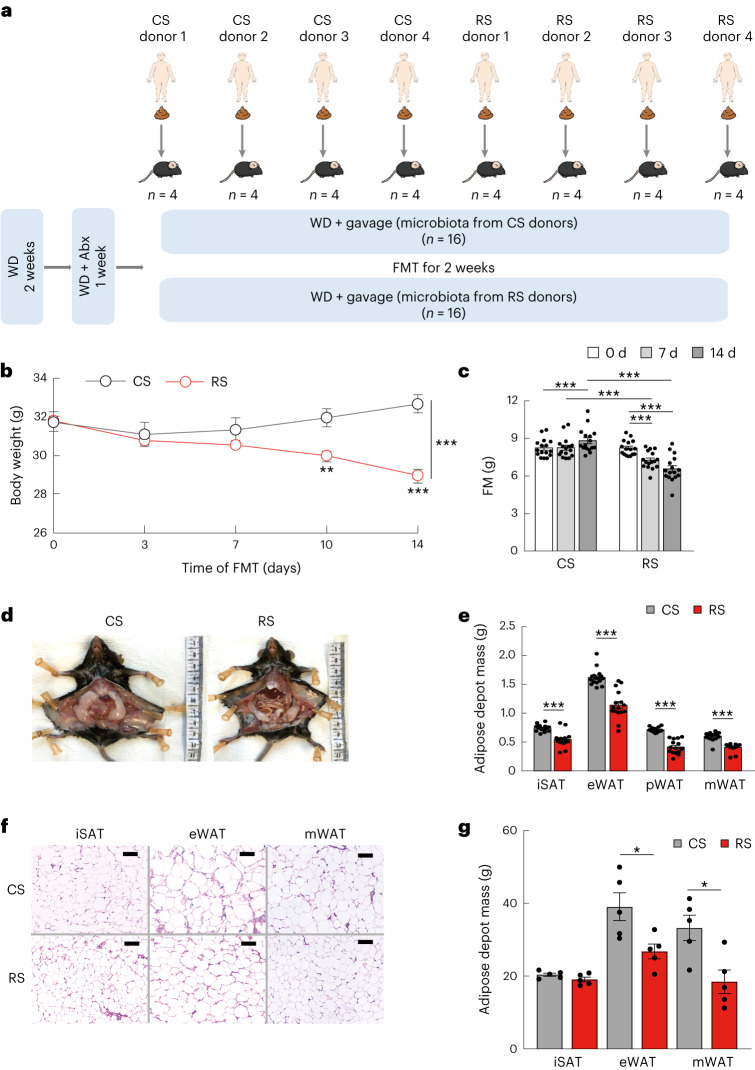
RS-influenced gut microbiota alleviates obesity. **a**, Schematic diagram of FMT. Faecal samples from human donors after an 8-week RS or CS intervention (*n* = 4 per intervention) were transplanted to antibiotic (Abx)-treated C57BL/6 mice. The mice were fed an irradiated western diet (WD) for 2 weeks before and during the 14 d of colonization (*n* = 16 per group). **b**,**c**, Body weight and fat mass changes after FMT. **d**, Representative photographs of visceral fat in mice colonized for 14 d with microbiota from RS or CS donors. **e**, Adipose depot mass including inguinal subcutaneous fat (iSAT), eWAT, pWAT and mWAT in mice colonized for 14 d with microbiota. **f**,**g**, Representative images and quantification data of haematoxylin and eosin-stained sections of iSAT, eWAT and mWAT in the two groups of mice colonized with CS (top) or RS microbiota (bottom). Scale bar, 100 µm. Data were reproduced in three independent experiments. Data are presented as mean ± s.e.m. Significance was determined by paired two-tailed Student’s *t*-test (**c**) and unpaired two-tailed Student’s *t*-test (**b**,**e**,**g**) (normally distributed) or nonparametric two-sided Wilcoxon rank-sum test (non-normally distributed). *P = 0.02 and 0.01 (**g**), **P = 0.002 (**b**), ***P < 0.001 (**b**,**c**,**e**). Source data

**Extended Data Fig. 7:**
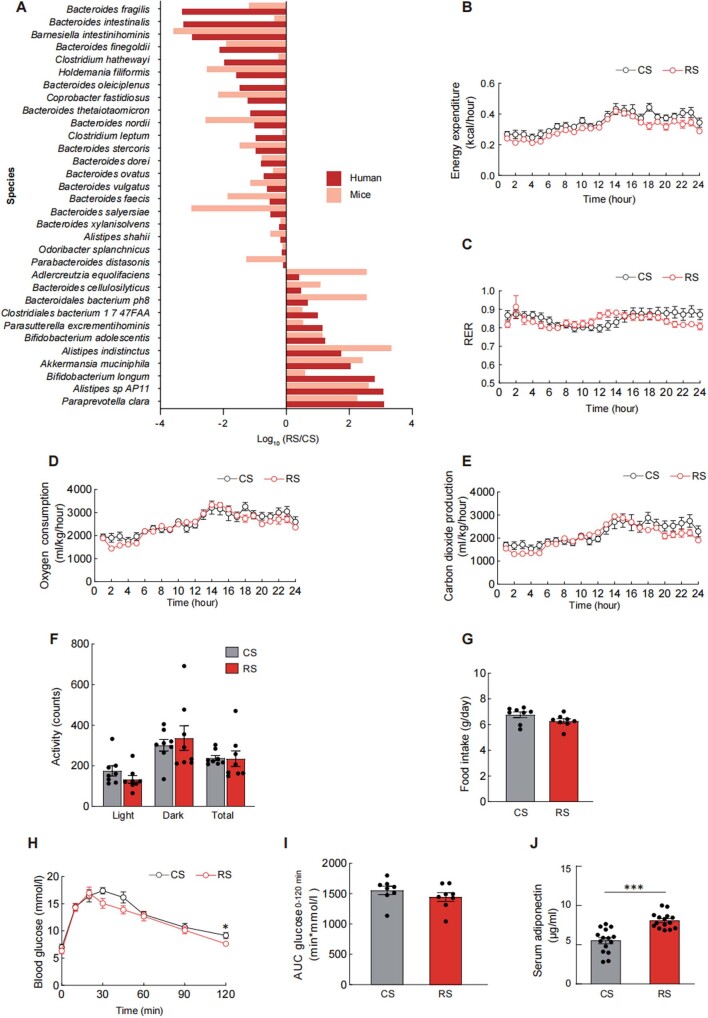
Change of microbial species, energy metabolism, and glucose metabolism after FMT. (**a**) Whole-genome shotgun metagenomic sequencing was performed for caecum content samples from mice receiving faecal slurry collected from donors collected after RS or CS intervention. Species showing a similar trend of RS-induced alterations between donors and mice receiving faecal microbial transplantation (FMT) were shown. Data are presented as log_10_ (RS/CS). RS/CS was defined as the ratio of relative microbial abundance after RS intervention (FMT from RS donors for mice) to that after CS intervention (FMT from CS donors for mice). (**b-i**) Mice were grouped and treated as in Fig. 4 (n = 8 biological replicates per group). (**b**) 24-hour energy expenditure, (**c**) respiratory exchange ratio (RER), (**d**) oxygen consumption, (**e**) carbon dioxide production, (**f**) activity, and (**g**) food intake were measured using the comprehensive laboratory animal monitoring system at the end of the experiment. (**h**) Glucose tolerance test (GTT) was performed in mice colonized with microbiota from RS or CS donors. (**i**) Area under the curve (AUC) analysis of GTT. (**j**) Serum adiponectin levels were measured at the end of faecal microbiota transplantation (n = 15 biological replicates per group). Data were reproduced in three independent experiments. Data are presented as mean ± s.e.m. Significance was determined by two-tailed Student’s t-test. * *P* = 0.03 (**h**), and *** *P* < 0.001 (**j**). Source data

To investigate the potential of the RS-related gut microbiota to trigger improvements in host abdominal obesity and glucose metabolism, we performed FMT in antibiotic-treated mice fed with a western diet, utilizing samples from human donors after RS or CS intervention (with changes in body weight post-intervention approximating the respective group average; *n* = 4 per group) (Fig. 4a). We observed a consistent trend of RS-induced alterations between donors and mice receiving FMT (Extended Data Fig. 7a). Two weeks after FMT, the body weight and fat mass of the mice that received the RS microbiota were lower than those of mice receiving the CS microbiota (*P* < 0.001) (Fig. 4b,c). The depot mass percentage of epididymal white adipose tissue (eWAT), peri-renal white adipose tissue (pWAT) and mesenteric white adipose tissue (mWAT) in mice colonized with the RS microbiota was significantly lower than that of mice colonized with CS microbiota (Fig. 4d,e). Histological analysis of eWAT and mWAT presented a substantially reduced size of adipocytes in mice colonized with the RS microbiota (Fig. 4f,g). A comprehensive laboratory animal monitoring system indicated that the two groups of mice did not show significant differences in energy consumption, respiratory exchange ratio, CO_2_ production, O_2_ consumption, activity and food intake (Extended Data Fig. 7b–g). The glucose tolerance in these mice was at least partially improved by transplanting the gut microbiota from humans after the RS intervention (Extended Data Fig. 7h,i). Circulating adiponectin levels in the mice colonized with the RS microbiota were significantly higher than in those colonized with the CS microbiota (Extended Data Fig. 7j). These results suggested that RS-induced changes in the gut microbiota could be sufficient to alleviate obesity and improve host glucose metabolism.

### RS-reshaped gut microbiota restores gut barrier

**Fig. 5:**
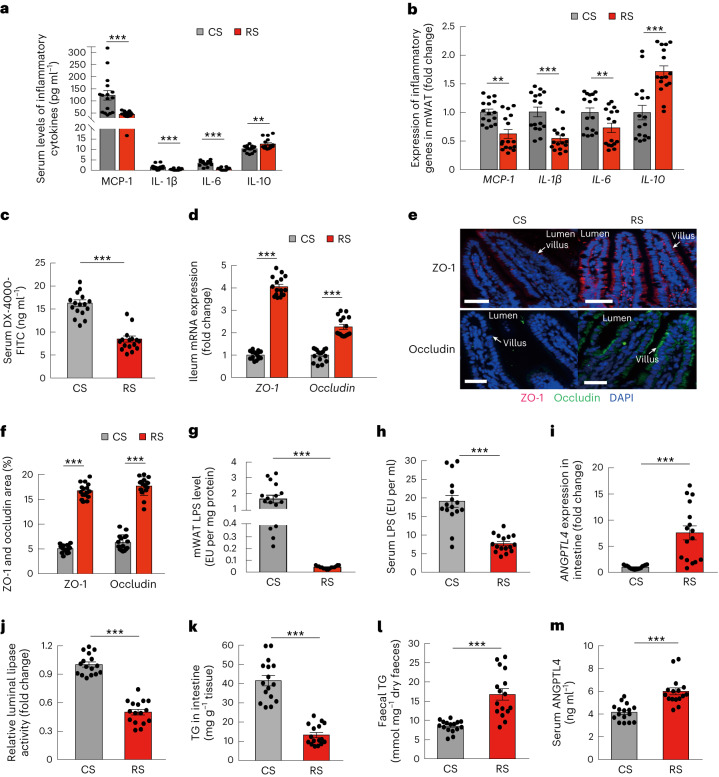
RS-influenced gut microbiota restores gut barrier and reduces lipid absorption. Mice were grouped and treated as in Fig. 4 (*n* = 16 per group). **a**, Serum levels of inflammatory cytokines in mice colonized with microbiota from RS or CS donors. **b**, The expression of inflammatory genes in mWAT and in mice colonized with microbiota from RS or CS donors. **c**, Gut permeability in vivo. **d**, The expression of *ZO-1* and *occludin* in the ileum. **e**, The localizations and levels of ZO-1 (red) and occludin (green) in intestinal villus were visualized by immunofluorescence and counterstaining with 4,6-diamidino-2-phenylindole (DAPI) (blue). Representative images of each group are shown (scale bar, 50 µm). **f**, Quantitative analysis of the positive stained area of ZO-1 and occludin was performed by ImageJ software and calculated as the percentage of total lesion area. **g**,**h**, LPS levels in mWAT and in circulation (serum). **i**, Expression levels of ANGPTL4 in the ileum. **j**, Relative intestinal luminal lipase activity. **k**, TG levels in the ileum. **l**, Faecal TG levels. **m**, Serum ANGPTL4 levels. Data were reproduced in three independent experiments. Data are presented as mean ± s.e.m. Significance was determined by unpaired two-tailed Student’s *t*-test (normally distributed) or nonparametric two-sided Wilcoxon rank-sum test (non-normally distributed). **P = 0.005 (**a**) and 0.001 and 0.010 (**b**), ***P < 0.001. Source data

Consistent with the clinical trial findings, RS-altered gut microbiota ameliorated systemic inflammation. Two weeks after FMT, circulating MCP-1, IL-1β and IL-6 levels in the mice colonized with RS microbiota were significantly lower than in those colonized with CS microbiota (all *P* < 0.01) (Fig. 5a). Serum levels of the anti-inflammatory factor IL-10 in mice colonized with RS microbiota increased (Fig. 5a). In mesenteric adipose tissues, the expression of *MCP-1*, *IL-1β* and *IL-6* was also significantly lower, whereas that of *IL-10* was higher in the mice receiving RS microbiota compared with those receiving CS microbiota (Fig. 5b).

Metabolic endotoxaemia, which may be attributable to the increased penetration of lipopolysaccharide (LPS) from the gut to circulation, serves as a pivotal factor in obesity-induced chronic inflammation^7^. To investigate how RS-altered gut microbiota interact with metabolic endotoxaemia, we further studied the in vivo gut permeability in mice colonized with microbiota from human donors following RS and CS interventions. The mice were orally gavaged with fluorescein isothiocyanate-labelled dextran (DX-4000-FITC) after a 6-h fasting period and subsequently, serum samples were collected via the tail vein. The concentration of DX-4000-FITC in the serum was determined using a fluorescence spectrophotometer. Intestinal permeability was significantly lower in mice colonized with RS microbiota compared with those colonized with CS microbiota (Fig. 5c). Gut permeability is regulated by intestinal tight junctions of that block the invasion of pathogens and bacterial products^7^. We evaluated the effect of the RS-induced gut microbiota changes on the expression levels of the major tight junction proteins in the intestine, zona occludens protein (ZO-1) and occludin. The expressions of both *ZO-1* and *occludin* genes were significantly increased in the mice that received the RS microbiota compared with those that received CS microbiota (Fig. 5d–f). Furthermore, in mice colonized with gut microbiota from donors after the RS intervention, restoration of the gut barrier led to a significant reduction in LPS levels in both mesenteric adipose tissue and circulation (Fig. 5g,h). These results suggested that one of the beneficial effects of the altered microbiota was to block LPS penetration by preserving the gut barrier.

### RS-altered gut microbiota reduces lipid absorption

Our clinical study showed significantly increased circulating levels of ANGPTL4 and faecal lipids in participants after RS consumption compared with CS consumption. To investigate whether the increase in ANGPTL4 was regulated by the RS microbiota, we measured the levels of *ANGPTL4* expression in the ileum of mice from the FMT experiments. Compared with mice colonized with CS microbiota, mice colonized with RS microbiota had significantly higher ileal *ANGPTL4* expression (Fig. 5i). ANGPTL4 is an endogenous inhibitor of lipoprotein lipase (LPL) and also pancreatic lipase, which functions as the principal intestinal lipase^32^. We found that the mice colonized with the RS microbiota had a significantly lower intestinal luminal lipase activity compared with mice that received CS microbiota (Fig. 5j). In accordance with the inhibited luminal lipase activity, mice colonized with RS microbiota exhibited lower TG levels in the ileum and higher TG levels in the faeces than mice colonized with CS microbiota (Fig. 5k,l). Circulating ANGPTL4 levels were also significantly increased in mice colonized with RS microbiota compared with mice colonized with CS microbiota (Fig. 5m). These results indicated that RS-induced changes in the gut microbiota were able to reduce lipid absorption through modulating intestinal ANGPTL4.

### Effects of *B.adolescentis* on obesity

**Fig. 6:**
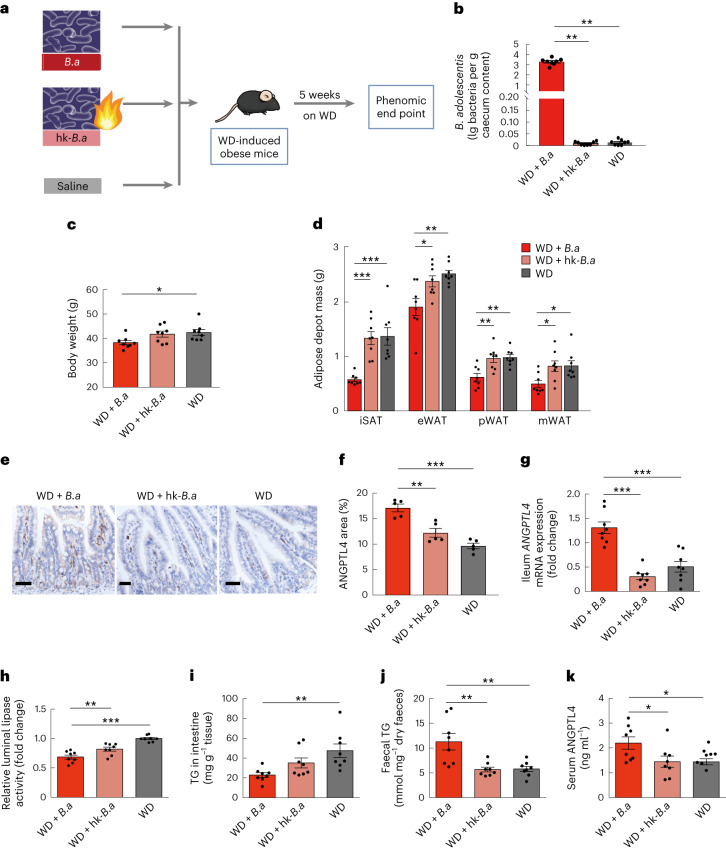
*B.* *adolescentis* protects against diet-induced obesity and affects intestinal and circulating ANGPTL4. **a**, Schematic diagram of *B.* *adolescentis* (*B.a*) supplementation strategy. WD-induced obese C57BL/6 mice were supplemented with drinking water with live (WD + *B.a*) or heat-killed *B.* *adolescentis* (WD + hk-*B.a*) or saline (WD) for 5 weeks. **b**, The abundance of *B.* *adolescentis* quantified by qPCR using the specific primers in caecum content among three groups. **c**–**k**, Parameters measured in the three groups after 5 weeks of treatment: body weight (**c**). Adipose depot mass including iSAT, eWAT, pWAT and mWAT (**d**). The localizations and levels of ANGPTL4 in ileum of mice were visualized by immunohistochemistry staining (scale bar, 100 µm) (**e**). Representative images of each group were shown. ANGPTL4^+^ area as a percentage of total ileal mucosa area (**f**). *ANGPTL4* mRNA expression levels in the ileum (**g**). Relative intestinal luminal lipase activity (**h**). TG levels in the ileum (**i**). Faecal TG levels (**j**). Serum ANGPTL4 levels (**k**). *n* = 8 biological replicates for each group (**b**–**d**,**g**–**k**). Data were reproduced in three independent experiments. Data are presented as mean ± s.e.m. Significance was determined by one-way ANOVA (normally distributed) followed by Tukey’s post hoc test or Kruskal–Wallis test (non-normally distributed) followed by Dunn’s test. *P = 0.04 (**c**), 0.02, 0.04, 0.03 (**d**) and 0.05 and 0.05 (**k**). **P = 0.001, 0.004 (**b**), 0.003, 0.004 and 0.002 (**d**), 0.001 (**f**), 0.009 (**h**), 0.002 (**i**) and 0.003 and 0.003 (**j**). ***P < 0.001. Source data

**Extended Data Fig. 8:**
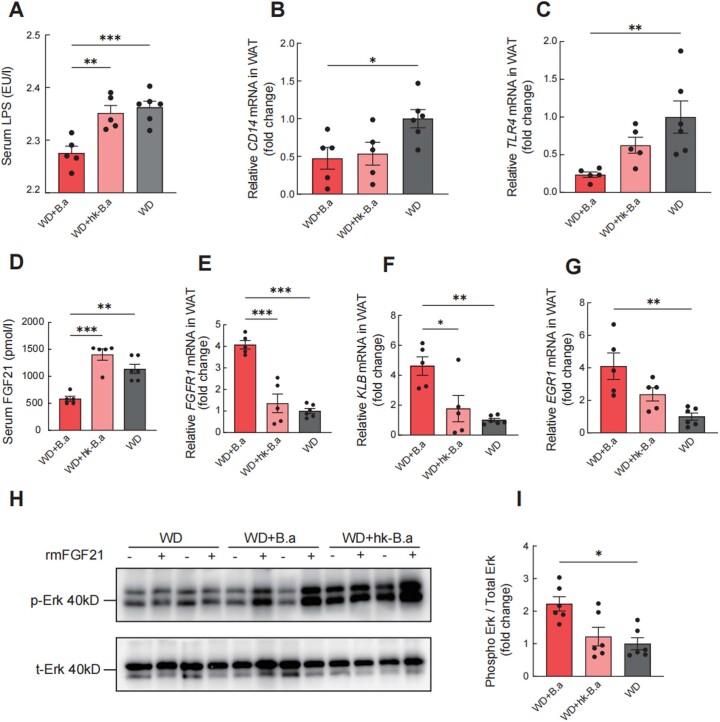
*Bifidobacterium adolescentis* enhanced the sensitivity of FGF21 in adipose tissue. The mice were fed the western diet for 8 weeks before administration of *B. adolescentis* and were randomized into three groups, drinking sterile water fortified with *B. adolescentis* (WD+B.a), heat-killed *B. adolescentis* (WD+hk-B.a) or PBS (WD) for 5 weeks. After an 8-week *B. adolescentis* intervention period, a fibroblast growth factor 21 (FGF21) response test was conducted on mice. (**a**) The level of serum lipopolysaccharide (LPS). (**b**) The transcription of cluster of differentiation 14 (CD14) in white adipose tissue (WAT). (**c**) The transcription of toll-like receptor 4 (TLR4) in WAT. (**d**) The serum level of FGF21. (**e**) The transcription of fibroblast growth factor receptor 1 (FGFR1) in WAT. (**f**) The transcription of βklotho (KLB) in WAT. (**g**) The transcription of early growth response factor 1 (EGR1) in WAT. (**h**) *In vivo* phosphorylation of extracellular signal-regulated kinase 1/2 (Erk1/2) (Thr202/Tyr204) and total Erk1/2 before and after rmFGF21 administration (2mg/kg, i.v.) detected by western blot analysis. (**i**) Quantification analysis of the fold change of Erk1/2 phosphorylation. The samples derive from the same experiment and that gels were processed in parallel. Data are mean ± s.e.m. WD+B.a, n = 5 biological replicates; WD+hk-B.a, n = 5 biological replicates; WD, n = 6 biological replicates. * *P* = 0.04 (**b**), 0.01 (**f**), and 0.02 (**i**), ** *P* = 0.004 (**a**), 0.009 (**c**), 0.001 (**d**), 0.002 (**f**), and 0.002 (**g**), *** *P* < 0.001, based on one-way ANOVA (normally distributed) followed by Tukey’s post hoc test or Kruskal–Wallis test (non-normally distributed) followed by Dunn’s test. Source data

The increase in *B.* *adolescentis* caused by RS was strongly correlated with the alleviation of abdominal obesity. Given this, we further investigated the potential causal association between *B.* *adolescentis* in the gut and host obesity in conventionally raised mice. Mice were randomized into three groups, drinking sterile water fortified with live *B.* *adolescentis*, heat-killed *B.* *adolescentis* or saline for 5 weeks (Fig. 6a,b). Supplementing drinking water with live *B.* *adolescentis* significantly alleviated body weight gain (Fig. 6c) and adiposity (Fig. 6d) in mice fed a western diet. We further investigated whether *B.* *adolescentis* regulated *ANGPTL4* expression in the intestine. In comparison with mice given drinking water supplemented with heat-killed *B.* *adolescentis*, ileal *ANGPTL4* expression and secretion were significantly higher in mice given drinking water supplemented with live *B.* *adolescentis* (Fig. 6e–g). Consistent with our clinical trial, *B.* *adolescentis* positively correlated with serum ANGPTL4 levels. Mice receiving live *B.* *adolescentis* had significantly decreased luminal lipase activity (Fig. 6h). Correspondingly, these mice had decreased ileal TG and increased faecal TG levels (Fig. 6i,j). Circulating ANGPTL4 levels were also significantly increased in the mice treated with live *B.* *adolescentis* (Fig. 6k). Our findings suggest that *B.* *adolescentis*, one of the key species induced by RS, may protect mice against diet-induced obesity by affecting intestinal ANGPTL4. In addition, the level of FGF21 significantly decreased in the participants after RS intervention, indicating the improvement of FGF21 sensitivity (Fig. 1m). We found that the treatment with *B.* *adolescentis* in mice increased the sensitivity to FGF21 in adipose tissue by suppressing the LPS–TLR4–NF-κB pathway (Extended Data Fig. 8).

**Extended Data Fig. 9:**
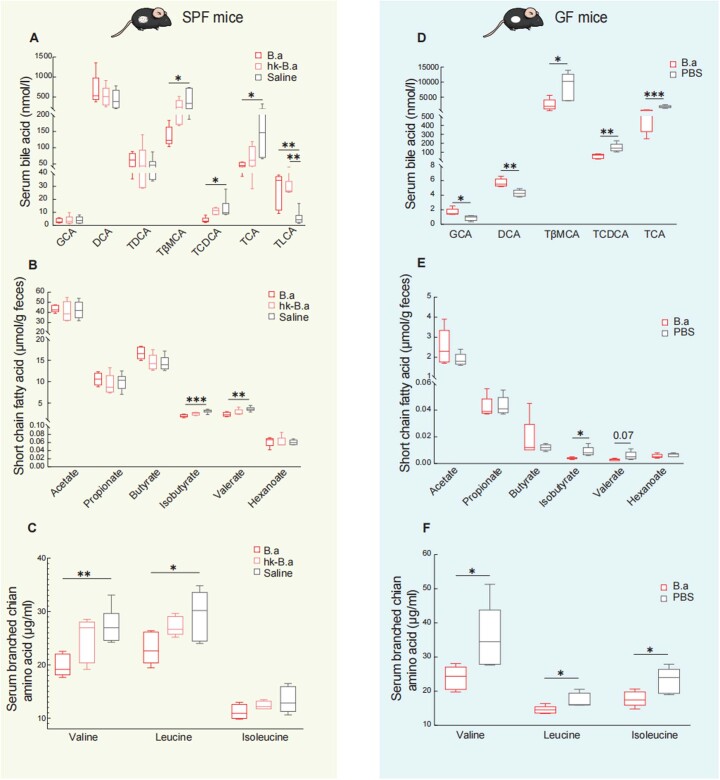
Supplementation with *Bacteroides adolescentis* altered metabolites in mice. Metabolomics profile of (**a-c**) SPF mice were grouped and treated as in Extended Data Fig. 7 (WD+B.a, n = 5 biological replicates; WD+hk-B.a, n = 5 biological replicates; WD, n = 6 biological replicates). and (**d-f**) germ-free mice were grouped and treated as in Fig. 7 (n = 5 biological replicates per group). (**a**) Levels of GCA, DCA, TDCA, TβMCA, TCDCA, TCA, and TLCA in serum. (**b**) Levels of SCFAs in faeces. (**c**) Levels of valine, isoleucine, and leucine in serum. (**d**) Levels of GCA, DCA, TβMCA, TCDCA, and TCA in serum of germ-free mice. (**e**) Levels of SCFAs in faeces of germ-free mice. (**f**) Levels of valine, isoleucine, and leucine in serum of germ-free mice. Data are presented as box-and-whisker plots. The horizontal line in the middle of each box indicates the median value, the top and bottom borders of the boxes represent the 75th and 25th percentiles, and the whiskers denote the lowest and highest values. Analysed by one-way ANOVA followed by Tukey’s post hoc test (in **a-c**) and two-tailed Student’s unpaired t-test (in **d-f**). * *P* = 0.05, 0.03, and 0.04 (**a**), 0.02 (**c**), 0.016 and 0.024 (**d**), 0.015 (**e**), and 0.034, 0.028, and 0.025 (**f**), ** *P* = 0.006 and 0.003 (**a**), 0.008 (**b**), 0.006 (**c**), 0.002 and 0.005 (**d**), *** *P* < 0.001. SPF, specific pathogen-free; GCA, glycocholic acid; DCA, deoxycholic acid; TDCA, taurodeoxycholate; TβMCA, tauro-β muricholic acid; TCDCA, taurochenodeoxycholic acid; TCA, taurocholic acid; TLCA, taurolithocholic acid. Source data

We further conducted a targeted metabolomics analysis in conventionally raised mice treated with *B.* *adolescentis* (Extended Data Fig. 9a–c). The primary bile acids, including tauro-β muricholic acid (TβMCA), taurochenodeoxycholic acid (TCDCA) and taurocholic acid (TCA) significantly decreased and the secondary bile acid taurolithocholic acid significantly increased after *B.* *adolescentis* intervention (Extended Data Fig. 9a). Regarding SCFAs, the faecal levels of isobutyrate and valerate also decreased significantly after *B.* *adolescentis* intervention (Extended Data Fig. 9b). In addition, the levels of serum branched-chain amino acids (BCAAs), including valine and leucine, significantly decreased after *B.* *adolescentis* intervention (Extended Data Fig. 9c).

### RS facilitates weight loss partially through *B.adolescentis*

**Fig. 7:**
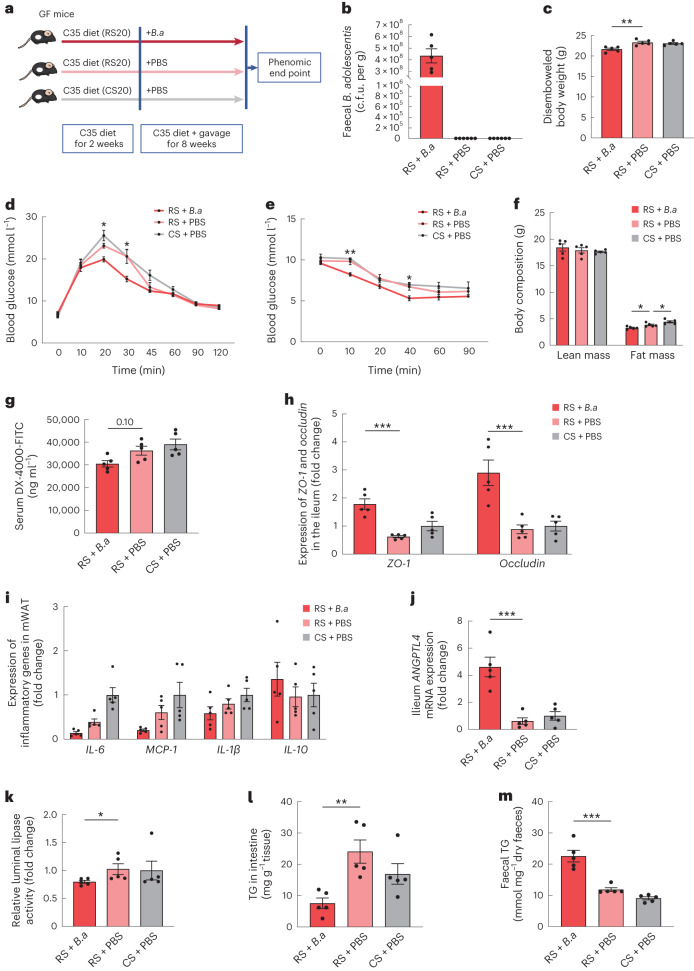
Effects of RS in germ-free mice with and without *B.* *adolescentis*. **a**, Schematic diagram of *B.* *adolescentis* (*B.a*) supplementation strategy. Orally inoculating *B.* *adolescentis* or PBS into germ-free (GF) mice on diets with 20% protein, 45% fat and 35% carbohydrate sourced (from 20% CS or 20% RS and the remaining 80% from maltodextrin) for 8 weeks. **b**, Abundance of *B.* *adolescentis* quantified as total plate count in caecum content. c.f.u., colony-forming unit. **c**, Disembowelled body weight. **d**, Glucose excursion curves of intraperitoneal glucose tolerance tests (GTTs). **e**, Glucose excursion curves of intraperitoneal insulin tolerance tests (ITTs). **f**, Body mass composition. **g**, After an 8-week *B.* *adolescentis* supplementation, in vivo gut permeability was determined by measurement of serum concentrations of DX-4000-FITC at 1 h after oral gavage. **h**, Expression of *ZO-1* and *occludin* in the ileum. **i**, Expression of inflammatory genes in mWAT. **j**, mRNA expression levels of *ANGPTL4* in the ileum. **k**, Relative intestinal luminal lipase activity. **l**, TG levels in the ileum. **m**, Faecal TG levels. Data are mean ± s.e.m. (*n* = 5 biological replicates per group). *P = 0.04 and 0.03 (**d**), 0.02 (**e**), 0.05, 0.04 (**f**) and 0.03 (**k**), **P = 0.002 (**c**), 0.002 (**e**), and 0.004 (**l**), ***P < 0.001 based on one-way ANOVA (normally distributed) followed by Dunnett’s test or Kruskal–Wallis test (non-normally distributed) followed by Dunn’s test. * indicates the comparison between RS + *B.a* and RS + PBS (**d**,**e**). Source data

To explore whether the effects of RS depend on gut microbiota, we carried out the RS and CS interventions in germ-free mice (Fig. 7a). Male germ-free mice were fed a C35 diet (35% carbohydrate sourced from 20% RS or 20% CS) for 10 weeks. Meanwhile, these mice were treated with PBS or live *B.* *adolescentis* for 8 weeks. As intestinal contents and the thickness of the lumen were significantly increased after RS feeding, the disembowelled body weight was commonly used to evaluate the effect of weight loss^12^. Unlike the protecting effect of RS against diet-induced obesity in conventional environment^33^, RS treatment showed no significant change on disembowelled body weight, glucose tolerance and insulin sensitivity compared with CS treatment in germ-free mice (Fig. 7c–f). To further investigated the effects of RS with and without *B.* *adolescentis*, we fed the germ-free mice with RS together with *B.* *adolescentis*. These mice showed significant decrease in disembowelled body weight and fat mass, as well as the improvement on glucose tolerance and insulin sensitivity compared with mice without *B.* *adolescentis* (Fig. 7c–f). *B.* *adolescentis* reduced gut permeability and increased the intestinal production of ANGPTL4 in germ-free mice, which was consistent with our finding in a conventional environment (Fig. 7g–k). Germ-free mice gavaged with *B.* *adolescentis* exhibited reduced luminal lipase activity and ileum TG levels, alongside increased faecal TG levels (Fig. 7l,m). These results indicated that gut microbiota was essential in the action of RS in protecting against diet-induced obesity. RS played the role in facilitating weight loss, at least partially, through *B.* *adolescentis* via reducing inflammation by restoring the gut barrier and modulating ANGPTL4 production in the intestine, thereby impeding lipid absorption.

In addition, the serum levels of primary bile acids in germ-free mice were higher than those in the conventional environment and the levels of secondary bile acids were relatively low^34^. GCA, a conjugated bile acid and DCA, a secondary bile acid, exhibit a tendency to increase after RS intervention in humans. GCA and DCA significantly increased after *B.* *adolescentis* intervention in germ-free mice. The primary bile acids, including TβMCA, TCDCA and TCA significantly decreased after *B.* *adolescentis* intervention in germ-free mice (Extended Data Fig. 9d). The levels of serum BCAAs and faecal isobutyrate were decreased after *B.* *adolescentis* intervention (Extended Data Fig. 9e,f).

## Discussion

**Extended Data Fig. 10:**
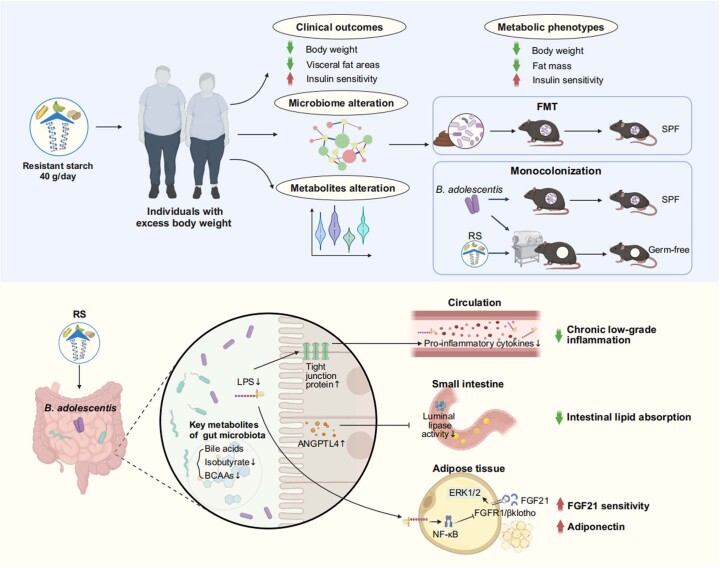
RS intake facilitates weight loss in humans by reshaping the gut microbiota. Resistant starch (RS, 40 g d^-1^) accompanied with isoenergetic and balanced diets led to an obvious reduction in body weight and improvement of insulin sensitivity, as well as alteration in metagenomics and metabolomics. Faecal microbiota transplantation (FMT) showed benefits of RS were associated with the reshaped gut microbiota composition. Monocolonization of mice with *B. adolescentis*, which was closely correlated with the benefits of RS in human protected mice from diet-induced obesity. Mechanistically, the RS-induced changes in the gut microbiota influenced metabolites of gut microbiome, reduced chronic low-grade inflammation by improving intestinal integrity, inhibited lipid absorption by modulating angiopoietin-like 4 (ANGPTL4), and improved the sensitivity of fibroblast growth factor 21 (FGF21) in adipose tissue. SPF, specific-pathogen-free; LPS, lipopolysaccharide; BCAAs, branched-chain amino acids; Erk1/2, extracellular signal-regulated kinase 1/2; FGFR1, fibroblast growth factor receptor 1. Created with BioRender.com.

This placebo-controlled, double-blinded and crossover-designed trial was conducted in individuals with excess body weight. We found that RS supplementation, coupled with isoenergetic and balanced diets, significantly reduced body weight and improved insulin sensitivity in humans. RS treatment reshaped the microbiome structure and altered metabolites. Gut microbiota plays a key role in RS’s efficacy for weight loss. Monocolonization of mice with *B.* *adolescentis*, linked to the benefits of RS in humans, prevented diet-induced obesity in mice. Mechanistically, the RS-induced changes in gut microbiota influenced bile acid metabolism, reduced inflammation through gut barrier restoration, inhibited lipid absorption by modulating ANGPTL4 and improved FGF21 sensitivity (Extended Data Fig. 10). RS played a role in facilitating weight loss at least partially through *B.* *adolescentis*. Specifically, this study provides evidence that RS (40 g d^−1^, type 2) as a dietary supplement for 8 weeks can help to achieve weight loss in individuals with excess body weight. In this feeding study, we provided the participants with a background diet throughout the study. The background diets, which were isoenergetic and balanced^20^, did not influence body weight or fat content over an 8-week period, as observed following CS treatment. In previous trials using RS, relatively insufficient RS intake (fat:fibre ratio ≈53:27 g d^−1^)^35^ or high fat intake (fat:RS ratio ≈77:40 g d^−1^)^14^ resulted in reduction of insulin resistance or fat percentage without weight loss. Other trials did not prescribe a background diet and the fat intake was either too high or not assessed^15,16^. High-fat diets induce changes in the intestinal microbiota that lead to impaired gut health^36^. In microbiome-based therapies, a background diet is critical for efficacy^7^. In rodents, low-fat diets (18% of energy) supplemented with RS have beneficial effects on the hosts, but high-fat diets (42% of energy) attenuated RS fermentation and the beneficial effects^18^. As 40 g d^−1^ RS was deemed to be a relatively high dose that could be delivered without adverse effects^37^, the fat content in the daily diet is important for the effect of RS. Our study provided an effective dietary recommendation using RS as a supplement (40 g d^−1^ with a balanced background diet containing 25–30% fat), which may help to achieve significant weight loss. The design of the background diet and good adherence enabled us to mitigate the influence of significant confounding variables known to have impacts on the gut microbiome and metabolome.

This study represents one of the few that reports a specific microbiota signature after RS2 intervention. The taxonomic groups commonly enriched post-RS2 consumption include *R.* *bromii*, *B.* *adolescentis*, *Faecalibacterium* *prausnitzii* and *Eubacterium* *rectale*. *R.* *bromii*, which acts as primary RS2 degrader was enriched during previous RS2 interventions^38^. In this study, *R.* *bromii* and *B.* *adolescentis* notably increased after RS intervention in individuals with excess body weight. The increased abundance of *B.* *adolescentis* strongly correlated with decreased BMI and VFA, suggesting its role in RS’s weight-loss benefits. Participants with *B.* *adolescentis* in their gut microbiome at baseline exhibited a greater decrease in fat mass after RS treatment. Furthermore, we transmitted the beneficial effects of RS on host obesity and glucose metabolism in mice via human microbiota engraftment, reinforcing our hypothesis that RS-induced changes in the microbiota can drive beneficial host outcomes. Furthermore, gut microbiota was essential in the benefits of RS, which was verified through RS intervention in germ-free mice.

The production of microbiota-derived metabolites is involved in the function of prebiotics^7^. Bile acids are significant signalling metabolites linking gut microbiota with the host^28^. Herein, the secondary bile acid, GDCA, was significantly increased after RS treatment, other secondary bile acids, such as DCA, 7-ketoLCA and TDCA, exhibited a tendency to increase. Secondary bile acids have been reported to ameliorate hepatic steatosis and augment insulin sensitivity^39–41^. BSH carries out bile acid deconjugation^31^. In our study, the abundance of the *BSH* gene decreased and showed a significant correlation with *B.* *vulgatus*. Inhibiting BSH activity emerged as a potentially beneficial approach to regulate lipid and energy homoeostasis, leading to an increase in conjugated bile acids^31,34^. In our cohort, conjugated bile acids, including GCA, GDCA and TDCA, demonstrated an increasing trend after RS treatment. Consistent with the findings in humans, GCA and DCA significantly increased after *B.* *adolescentis* intervention in germ-free mice. GCA and DCA are the agonists to FXR and Takeda G protein-coupled receptor 5 (TGR5), respectively, which are known receptors regulating glucose, lipid and energy metabolism^42^. The serum level of BCAAs, including valine and leucine, significantly decreased after *B.* *adolescentis* intervention in both conventional and germ-free environment. Serum levels of BCAAs decreased after the RS intervention in participants with non-alcoholic fatty liver disease (NAFLD)^43^. Faecal isobutyrate, a microbial product of the proteolysis of valine^44,45^, decreased after RS intervention in our study.

The interplay between energy regulation and inflammation was modulated by the gut microbiome, influencing intestinal permeability and generating pro-inflammatory factors that exhibited variable effects on metabolic health^7^. Integrity of the intestinal mucosa is maintained via intercellular tight junctions, which are essential regulators of intestinal permeability^22^. The levels of the two tight junction proteins, occludin and ZO-1, elevated in the ileum and circulating LPS decreased in mice that received RS-induced microbiota, suggesting an additional mechanism of protecting the intestinal barrier by RS-altered gut microbiota. Chronic low-grade inflammation and immune system activation were involved in the development of obesity and insulin resistance^22^. Weight management influenced by gut microbiome may also through modulation of secretory protein involved in energy utilization and homoeostasis^7^. Notably, the metagenomics analysis demonstrated that the increased abundance of *B.* *adolescentis* after the RS treatment was positively associated with serum ANGPTL4. ANGPTL4 has garnered substantial interest in relation to the gut microbiota. Germ-free mice exhibited elevated intestinal *ANGPTL4* expression in compared with mice raised conventionally^3^. The reduction in ANGPTL4 levels contributes to increased fat accumulation upon conventionalization through increased LPL activity in adipose tissue^3^. Colonization of germ-free mice with acetate-producing *Bacteroides* *thetaiotaomicron* or methane-producing *Methanobrevibacter* *smithii*, resulted in decreased intestinal *ANGPTL4* expression, whereas colonization with the butyrate-producing bacteria *Clostridium* *tyrobutyricum* significantly elevated intestinal *ANGPTL4* expression^46^. A possible explanation for this result is that the modulation of intestinal ANGPTL4 is contingent on the gut microbiota composition, which determines the mixture of microbial metabolites formed, together with substrate provision^46^. Using FMT, we observed that RS-induced changes in gut microbiota reduced lipid absorption through modulating intestinal ANGPTL4. Furthermore, colonization of diet-induced obese mice raised in both conventional and germ-free environments with *B.* *adolescentis* markedly elevated intestinal ANGPTL4 levels. Thus, manipulating pancreatic lipase activity via ANGPTL4, a key regulator of lipid absorption, through gut microbiota modulation, might be a crucial way to alter body fat storage. *B.* *adolescentis* supplementation could mitigate NAFLD by enhancing FGF21 sensitivity in the liver^47^. Our results also revealed that treatment with *B.* *adolescentis* increased FGF21 sensitivity in adipose tissue by suppressing the LPS–TLR4–NF-κB pathway. Multiple clinical trials of FGF21 analogues and mimetics showed improved lipid profiles, increased adiponectin and decreased body weight^24^. It was uncovered that the reversal of obesity-induced FGF21 resistance in adipose tissue could serve as an alternative approach for treating obesity and related diseases^48^.

The main limitation of the study was its relatively small sample size and stringent inclusion criteria for participants, limiting result generalizability. Our database-dependent, taxon-based analysis, while offering comprehensive and well-annotated information for taxonomic assignments, also faces limitations such as discarding sequences that are difficult to classify and overlooking strain-level functional diversity. We acknowledge that integrating metagenome-assembled genomes could enhance our understanding of microbial ecology and potentially improve the identification of robust biomarkers for clinical translation. The broader generalizability of our results requires additional validation in larger and more diverse cohorts. Future studies will need to pay special attention to the individual dynamics and functional responses of microbiota in RS supplementation. The in-depth insight into the crosstalk between RS-altered bile acids and gut microbiota and its impact on host metabolism are warranted in further investigations. Although the applicability to other populations remains unconfirmed, our study provides the evidence that RS (40 g d^−1^, type 2) as a dietary supplement facilitates weight loss in individuals with excess body weight. Our study also provides an effective dietary design along with RS to mitigate the influence of major confounding variables affecting the gut microbiome. Gut microbiota change driven by RS was essential for its effect. The gut microbiota played a pivotal role in this weight-loss mechanism, potentially modulating obesity through interactions with low-grade inflammation and regulation of energy-balance-related secretory proteins. Through the crossover design, we found that body weight was regained during the washout period. Weight regain is one of the biggest challenges of weight loss treatment, which occurs after stopping medication, such as GLP-1 agonists and even after bariatric surgery^49^. Long-term adherence to an RS-rich dietary pattern to maintain the composition of the microbiome may be crucial for weight maintenance. As RS occurs naturally in foods and can also be added to daily diets, our findings provide a pragmatic lifestyle to treat obesity and its related metabolic disorders. Manipulating the gut microbial composition through diet may represent a strategy for modifying host energy balance to promote health.

## Methods

### Study participants

The inclusion criteria were (1) age 18–55 years and (2) overweight or obesity, as defined as BMI ≥ 24 kg m^−2^ or waist circumference ≥85 cm in men and ≥80 cm in women^20^. The exclusion criteria were acute illness, antibiotic or probiotic supplement use within 3 weeks previously, diagnosis of hyperthyroidism or hypothyroidism, diabetes, current systemic corticosteroid treatment or medications affecting glucose metabolism, and participation in other clinical trials within 4 weeks before the study.

This study was approved by the ethics committee of the Shanghai Sixth People’s Hospital and conformed with the Declaration of Helsinki. Written informed consent was obtained from all participants. Complete clinical trial registration was deposited in the Chinese Clinical Trial Registry (ChiCTR-TTRCC 13003333) (Extended Data Fig. 1).

### General protocol of the clinical trial

The detailed study protocol was submitted to the Chinese Clinical Trial Management Public Platform and is available in the Supplementary Information. Individuals with excess body weight consumed either RS derived from maize (HAM-RS2, Hi-Maize 260 resistant starch, 22000B00, provided by Ingredion) or energy-matched CS (AMIOCA cornstarch, 04400110, also provided by Ingredion) alternately for 8 weeks and separated by a 4-week washout period. An independent researcher performed the randomization and participant allocation to the CS-Washout-RS or RS-Washout-CS intervention schemes with at a 1:1 allocation ratio. The randomization schedule was produced by SAS PROC PLAN in SAS software. RS and CS were packaged in identical sealed bags with an identical appearance and participants and investigators were blinded to the group allocations during the double-blind period. Only the research designer was aware of the randomization scheme, whereas the participants, investigators, clinical staff and outcome assessors were blinded to it. The blinding was lifted during the bioinformatics analysis to explore the potential mechanism by which the gut microbiota conferred the physiological benefits of RS.

During this feeding study, participants received a uniform background diet, following the Chinese and American guidelines for the prevention and control of adults with overweight and obesity^20,21^. All participants were either lightly active or led sedentary lives. The diet provided 25 kcal kg^−1^ ideal body weight (ideal body weight (kg) = height (cm) − 105) daily, with 50–60% carbohydrate, 25–30% fat and 15–20% protein. Participants were permitted one item of fruit daily and advised against extra-sugary beverages or snacks. They maintained a diary recording three consecutive 24-h dietary intake (two weekdays and one weekend day) at baseline and at the beginning and end of each intervention period, noting any dietary deviations. A trained dietician assessed starch consumption and dietary regimen adherence during visits. Diet data revealed similar average calorie intake and macronutrient percentages in the RS and CS intervention periods (Supplementary Table 1).

Participants attended the institute for visits (V) 1–10 (Fig. 1a). At the start and the end of each intervention (V1, V5, V6 and V10), participants had a hyperinsulinemic–euglycemic clamp, MTT, MRI scans and sample collections^37^. Anthropometric and biochemical assessments were conducted at each visit. The primary outcome was body weight and secondary outcomes included VFA and SFA, body fat, waist circumference, lipid profiles, insulin sensitivity, metabolome and gut microbiome.

### Anthropometric and biochemical assessments

After overnight fasting for at least 10 h, anthropometric test and samples (venous blood, urine and faeces) collection was conducted in the morning following the study protocol.

Serum samples after centrifugation were stored at −80 °C until measuring AST, ALT, GGT, HDL-C, LDL-C and insulin^37^. Serum A-FABP (AIS, HKU, 31030), adiponectin (AIS, HKU, 31010), inflammatory cytokines (Ebiosciense, TNFα, BMS223HS; MCP-1, BMS281; IL-1β, BMS224HS; IL-6, BMS213HS; IL-10, BMS215HS), ANGPTL4 (BioVendor, RD191073200R) and FGF21 (AIS, HKU, 31180) were quantified by enzyme-linked immunosorbent assay (ELISA). Serum LPS levels were measured through sensitive limulus amoebocyte lysate (LAL) assay (Hycult Biotechnology, MAK109).

### MTT experiment

To evaluate glucose metabolism, serial blood samples were collected in both fasting and postprandial states for laboratory tests following a standard meal (315.2 kcal, including 68.4 g carbohydrate and 10.4 g protein) (China Oil & Foodstuffs Corporation).

### Hyperinsulinemic–euglycemic clamp

Insulin sensitivity was evaluated through a hyperinsulinemic–euglycemic clamp. Insulin levels were maintained at approximately 100 μU ml^−1^ through a prime-continuous infusion of insulin. To maintain the concentration of plasma glucose at basal levels, variable glucose infusion following a negative feedback principle was conducted. The glucose clamp technique was performed as previously described^50^.

### Faecal DNA extraction and sequencing

Faecal samples were collected through a tube with a DNA stabilizer (STRATEC Molecular) and stored at −80 °C. Faecal genomic DNA from both humans and mice receiving FMT from human donors was extracted using PSP Spin Stool DNA kit (STRATEC Molecular, 1038100) following the manufacturer’s instructions. Faecal DNA extracts from 86 samples (27 samples before and after RS and 16 samples before and after CS) were randomly selected from all participants for shotgun metagenomic sequencing. HiSeq 1500 was used for 100-bp paired-end shotgun metagenomic sequencing for human samples at the Centre for Genomic Sciences, the University of Hong Kong. An Illumina platform Novaseq 6000 was used for 150-bp paired-end sequencing for mice samples at Novogene. Raw sequences were stored in the National Center for Biotechnology (NCBI) Sequence Read Archive (project ID PRJNA414688).

### Metagenomics analysis

#### Quality control for metagenomic data

We used a series of quality control steps to remove low-quality reads/bases as described previously^51^. In brief, all Illumina primer/adaptor/linker sequences were removed and low-quality regions (quality score <20) and reads were trimmed. Subsequently, we mapped all reads to the human genome using BWA v.0.7.4 (ref. ^52^). Reads with >95% identity and 90% coverage were removed as human DNA contamination.

#### Taxonomy and functional profiling

Taxonomy affiliations for reads were determined by MetaPhlAn^53^. We distilled the top 50 most-abundant species based on the average abundance across all samples (minimum average abundance > 0.3%), which accounted for 91% of the total relative abundance in each sample on average. The proportion of unclassified reads was 8.06 ± 6.71% (mean ± s.d.) across all samples. To overcome potential bias from baseline differences between individuals within groups and to facilitate cross-group comparisons, we compared community-level variation patterns (based on the fold change in abundance of species), instead of raw community structures between two groups (based on the relative abundances of species). We calculated the log_2_FC in abundance after treatment for each species. Fold change values were normalized (within all samples in each group) ranging from 0 to 1 and further rescaled to make the sum of the normalized fold change as one. We further calculated the Bray–Curtis distance based on the profile of the normalized fold change of species abundance.

Differentially varied species after treatment with RS or CS were tested using a Wilcoxon signed-rank test, with an FDR cutoff of 0.2. The NMDS ordination analysis based on the normalized Bray–Curtis dissimilarity of species-level abundance fold change, described above, was performed with R package VEGAN^54^. Network visualization was performed by Cytoscape 3 (ref. ^55^).

IDBA-UD^56^ was adopted de novo assemblies with *k*-mer size ranging from 20 to 100 bp. The method for functional annotation and abundance calculations for KEGG pathways is described in an online server that we developed^57^. In brief, MetaGeneMark^58^ was used for microbial gene prediction. Metagenomic reads were mapped to assembled contigs to get gene-level DNA abundance levels with reads per kilobase per million (RPKM) mapped reads. We used KOBAS^59^ to annotate KEGG orthologues and pathways based on annotated genes. Scripts from our publish servers^57^ were used to calculate the aggregated RPKM for each pathway.

#### Metagenomics association analyses

Generalized linear modelling was performed using the R package glmulti. For each phenotype, the response variable was the fold change of the phenotype; the independent variables were from a subset of the fold change of 12 species with FDR < 0.3 in the differentially varied analysis using a Wilcoxon rank-sum test. The linear models started from ‘PHENOTYPE ~’, generating all possible models to reach a best model with feature selections. The importance for a particular species variable was calculated as the proportion of the total weights/probabilities for the models containing the variable during automatic model selection based on corrected AIC. This importance index reflects overall support of all possible weighted linear models. Only the main effects in the generalized linear models were considered. The *P* value of a particular species variable was calculate using Fisher’s method to combine the *P* value among the top ten models.

To quantify the association between (1) phenotypes and metabolites, (2) gut microbes and blood metabolites and (3) gut microbial pathways and blood metabolites, we adopted partial least-squares discriminant analysis (PLS-DA) using the mixOmics package^60^ in R. The number of components was set to 2 and number of features for each component was estimated based on hyperparameter optimization.

### Metabolomics profiling of human serum samples

The serum untargeted metabolomics data were quantified through a Shimadzu Prominence HPLC system (Shimadzu) coupled to an LTQ Orbitrap Velos instrument (Thermo Fisher Scientific) at the CAS Key Laboratory of Separation Science for Analytical Chemistry. The details of analytical conditions were as described in a previous study^61^. The quantitation of bile acid profiles was performed by Metabo-Profile^62–64^.

### Metabolomics profiling of human faecal samples

The metabolomics data of human faecal samples were quantified by GC–TOF–MS (Agilent 6890N GC coupled with a LECO Pegasus HT TOF–MS). The details of analytical condition as described in previous study^65^.

### Faecal lipid assessment

Faecal lipids were extracted from the faeces of participants after 8-week RS or CS interventions using published methods^66,67^. Approximately 100 mg lyophilized faeces was acidified with 100 μl ethanol and 500 μl 8 N hydrochloric acid, then kept in a water bath at 80 °C for 40 min. Fatty acids were subsequently extracted with 1,250 μl diethyl ether and 1,250 μl petroleum ether. After filtering the ether-fat upper phase, the clear ether solution was collected and kept in a rotary evaporator for 1 h at 40 °C. Fatty acids were resolubilized in ethanol and NEFA, TC and TG were subsequently measured using enzymatic assays (Jiancheng, A042-1-1, A111-1-1 and A110-1-1, respectively).

### Mouse model

The Committee on the Use of Live Animals for Teaching and Research of the University of Hong Kong and the Animal Ethics Committee of Shanghai Sixth People’s Hospital approved the animal experimental procedures. Healthy C57BL/6J male mice were conventionally raised in a specific-pathogen-free barrier facility in individually ventilated cages with a maximum of four mice per cage, with free access to food and water under a strict 12-h light–dark cycle at a controlled temperature (23 ± 2°C) and 60–70% humidity. For FMT experiments, 10-week-old male C57BL/6J antibiotic-treated mice (GemParmatech, N000013) were fed a WD (Research Diet, D12451) for 2 weeks before and during the colonization. For administrating of *B.* *adolescentis* to conventionally raised mice, eight-week-old males were similarly fed the WD for 8 weeks before *B.* *adolescentis* administration. For the intervention of RS with *B.* *adolescentis* in mice housed in a GF environment, 5-week-old male C57BL/6J mice were kept in isolators (GemParmatech, N000295) and fed a C35 diet (45% fat, 20% protein and 35% carbohydrate sourced from 20% RS or 20% CS, and the remaining 80% from maltodextrin) for 10 weeks. Meanwhile, the mice were orally inoculated with *B.* *adolescentis* or PBS for the remaining 8 weeks.

### FMT experiment

Antibiotic-treated mice with microbiota depletion were given daily oral gavage with a 200-µl antibiotics cocktail (ampicillin 1 g l^−1^, neomycin 1 g l^−1^, metronidazole 1 g l^−1^ and vancomycin 0.5 g l^−1^) for 7 d, followed by a 4-d antibiotic washout period before FMT. For FMT, the preparation of fresh stool samples and the subsequent operation in mice was conducted as previously described^43^.

Body weight was measured every 3 or 4 d. Stool samples were collected before and after faecal transplantation and instantly stored at −80 °C until further analysis. Body composition was determined with a Minispec LF90 Body Composition Analyzer (Bruker). In vivo gut permeability, GTTs, ITTs and whole-body oxygen consumption were accessed via a comprehensive laboratory animal monitoring system (Columbus Instruments)^68^. Fat mass in inguinal subcutaneous, epididymal, peri-renal and mesenteric white adipose tissue was determined after death by wet weight measurements. Blood and various tissues were collected for further biochemical evaluations. The study did not blind investigators to group allocations and no mice were excluded.

### Bacterial strain

*B.* *adolescentis* strain E 298 b (Variant c) (DSMZ no. 20086) was purchased from DSMZ. The culture medium was prepared according to the DSMZ website and *B.* *adolescentis* was grown in an anaerobic workstation (Gene Science AG300). *B.* *adolescentis* was cultured anaerobically in sterilized DSMZ Medium 58 containing casein peptone (tryptic digest, Sigma-Aldrich), yeast extract (Sigma-Aldrich), meat extract (Sigma-Aldrich), bacto soytone (BD), glucose (Sigma-Aldrich), K_2_HPO_4_, MgSO_4_ × 7 H_2_O, MnSO_4_ × H_2_O, Tween 80, NaCl, cysteine-HCl × H_2_O (Sigma-Aldrich), salt solution and 0.025% resazurin (Sigma-Aldrich). The purity of cultures was monitored by Gram staining and the c.f.u. was counted by plating serial dilutions on agar plates.

### Administration of *B.adolescentis*

*B.* *adolescentis* was collected through centrifugation at 1,500*g* for 30 min at 4 °C, followed by a double washing with sterile PBS and subsequently resuspended in 2 ml anaerobic sterile PBS containing 20% glycerol, achieving of 5 × 10^11^ c.f.u. per ml. This suspension was then preserved by storage at −80 °C. Before supplementing the drinking water of mice, live *B.* *adolescentis* stock was thawed at 4 °C and diluted in 100 ml autoclaved water. On average, each mouse received at least 4 × 10^10^ c.f.u. per day. *B.* *adolescentis* was heat-killed at 121 °C under pressure of 225 kPa for 15 min. Viability confirmation tests were performed by culturing, showing that heat-killed *B.* *adolescentis* did not grow, whereas live *B.* *adolescentis* grew well. Drinking water was changed daily in the three groups during the experiment. In addition, GF mice were treated with PBS or 1 × 10^9^ c.f.u. of live *B.* *adolescentis* in 200 μl sterile anaerobic PBS by gavage three times per week for 8 weeks.

### Biochemical and immunological assays in mice

Serum levels of TC and TG as well as intrahepatic, intestinal and faecal TG were measured by enzymatic analysis (Stanbio Laboratory, 2100430 and 1100430). Serum NEFA was measured by enzymatic analysis (Roche Diagnostics). Serum adiponectin (AIS, HKU, 32010), ANGPTL4 (Abcam, ab210577), inflammatory serum molecules (eBioscience, MCP-1, BMS6005; IL-1β, BMS6002; IL-6, BMS603-2; IL-10, BMS614INST) and FGF21 (Immunodiagnostics Limited, 32180) were measured using ELISA. LPS levels in mesenteric white adipose tissue and serum were measured by LAL assay (Hycult Biotechnology, HIT302).

### Metabolomics profiling of mice samples

#### Amio acid profiling

Target amino acids were quantified using Waters AccQ-Tag derivation kit with 20 μl serum by A Nexera X2 ultra HPLC equipped with a triple quadrupole 8050 (Shimadzu). The details of analytical condition as described in previous study^61^.

#### Bile acid profiling

Target bile acids were analysed by A Vanquish UPLC-Q Exactive (Thermo Fisher Scientific) system. Bile acids in THE serum sample (50 μl) was extracted by 200 μl methanol containing internal standards. Peak detection and integration were carried out by using Trancefinder (Thermo Fisher). The results quantified by the standard curve were used for feature analysis. The details of sample preparation and analytical conditions are as described in a previous study^69^.

#### SCFA profiling

For sample pretreatment, 30 μl serum was mixed with 60 μl acetonitrile containing the internal standard 0.3 μg ml^−1^ butyric acid-d7. After vortexing and centrifuging, the supernatant was aspirated for subsequent analysis. For the faecal sample, 40 mg faecal with grinding ball was mixed with 200 μl of acetonitrile containing internal standard extractant, internal standard Butyric-d7 acid 20 μmol l^−1^. After grinding and centrifugation, the upper supernatant was aspirated for subsequent analysis.

For analytical measurement, an Agilent 5977A instrument equipped with an MSD gas chromatography mass spectrometry (GC–MS) system (Agilent) was used for SCFA analysis. A DB-FFAP column (30 m × 250 μm × 0.25 μm, Agilent) was adopted for GC separation. The initial temperature was 50 °C and maintained for 1 min, then increased to 180 °C at 10 °C min^−1^, immediately raised to 250 °C at 30 °C min^−1^ and maintained for 2 min. The flow rate of helium carrier gas was kept at 40 cm s^−1^. The temperatures of the injector, ion source, quadrupole and interface were 250, 230, 150 and 250 °C, respectively. The selected ion monitoring mode was performed by detecting the protonated molecules at *m*/*z* 43 for acetic acid and isobutyric acid, *m*/*z* 74 for propionic acid and *m*/*z* 60 for other SCFAs. Peak detection and integration were performed using Quantitative Analysis (Agilent). The results quantified by the standard curve are used for feature analysis. All the metabolomic profiles in mice were performed at the CAS Key Laboratory of Separation Science for Analytical Chemistry.

### FGF21 response test

After an 8-week *B.* *adolescentis* intervention period, an FGF21 response test was conducted in conventionally raised mice. Under general anaesthesia, a subcutaneous fat biopsy was taken and flash-frozen in liquid nitrogen. Subsequently, 2 mg kg^−1^ recombinant mouse FGF21 or a vehicle was intravenously injected via the inferior vena cava. At 15 min later, a second subcutaneous fat biopsy was taken to assess gene expression and Erk1/2 phosphorylation.

### Quantitative PCR assays

The total RNA of tissue samples was extracted by TRIzol (Invitrogen) and reverse transcribed into complementary DNA using ImProm-II reverse transcriptase (Promega). Quantitative PCR with reverse transcription (RT–qPCR) reactions were performed using the Quantifast SYBR Green master mix (QIAGEN) on a Light Cycler 480 system (Roche), normalized with mouse GAPDH. Primers for each gene and *B.* *adolescentis*^70^ are listed in Supplementary Table 11.

### Histological examination

Ileum sections were treated with ZO-1 or occludin antibodies (Abcam, 1:500 dilution, ab96587; 1:100 dilution, ab216327), followed by incubation with Alex Fluor-596 or FITC-conjugated secondary antibodies (Life Technologies) and counterstaining with DAPI. Ileum sections were treated with ANGPTL4 antibody (Proteintech, 1:500 dilution, 18374-1-AP), followed by incubation with horseradish peroxidase-labelled secondary antibodies (DAKO), developed by DAB following the manufacturer’s recommendations. Five random fields were selected from different regions of individual sections for image quantitation. ImageJ software was used to analyse the intensity of positive staining, represented as a percentage of the total area of the lesion or villi in every field.

### Luminal lipase activity

The mixture of luminal content from the small intestine and ice-cold PBS was vortexed thoroughly, followed centrifugation at 15,000*g* at 4 °C for 10 min. Supernatants were collected and diluted 1:100 in PBS for lipase activity measurement via lipase activity assay kit (Sigma, MAK109) following the manufacturer’s protocol.

### Western blotting

WAT proteins were extracted using a lysis buffer containing protease inhibitors (ST505, Beyotime Biotechnology) and phosphatase inhibitors (P1082, Beyotime Biotechnology). Protein samples were resolved by SDS–PAGE (8%) and immunoblotted onto polyvinylidene difluoride membranes. Immunoblotting was conducted using extracellular signal-regulated protein kinase 1/2 (Erk1/2) (Cell Signalling Technology, 1:1,000 dilution, 9102) and phosphorylated Erk1/2 (Thr202/Tyr204) (Cell Signalling Technology, 1:1,000 dilution, 9101). Immunoreactivity was detected using enhanced chemiluminescent autoradiography (Millipore). Band quantification was performed using ImageJ software.

### Statistical analysis

The statistical analysis plan for this study is available in the Supplementary Information. EpiData v.3.1 facilitated clinical data entry and documentation. Statistical analyses unless otherwise stated, utilized SPSS v.17 and R v.3.3.2 software. Normally distributed clinical data were presented as mean ± s.d. Non-normally distributed data, verified by the Kolmogorov–Smirnov test, were presented as medians and IQRs. The intervention’s effects on outcomes were analysed using a linear mixed model adjusted for age, sex and intervention order, with Bonferroni adjustment for multiple testing correction. In animal studies, data distribution and variance equality were assessed using a Shapiro–Wilk test and Levene’s test. For two-group comparisons, a two-tailed Student’s unpaired *t-*test (normally distributed) or nonparametric Wilcoxon rank-sum test (non-normally distributed) was applied. Among multiple groups, a one-way ANOVA (normally distributed) followed by Tukey’s post hoc test or Kruskal–Wallis test (non-normally distributed) followed by Dunn’s test was used. In the clinical study, based on literature and preliminary data, the expected change in body weight and its s.d. after RS intervention was estimated as 2 ± 2.8 kg. A two-tailed test with a 0.05 significance level and 80% power required a minimum sample size of 31. Thirty-seven participants were invited to account for an expected 17% dropout post-randomization. For animal studies, sample sizes were estimated based on previous research and assay variability. Two-sided *P* values < 0.05 were deemed statistically significant.

### Reporting summary

Further information on research design is available in the Nature Portfolio Reporting Summary linked to this article.

### Supplementary information

Supplementary Information Study protocol and statistical analysis plan.

Reporting Summary

Supplementary Table 1 Supplementary Tables 1–11.

### Source data

Source Data Fig. 2 Statistical Source Data.

Source Data Fig. 3 Statistical Source Data.

Source Data Fig. 4 Statistical Source Data.

Source Data Fig. 5 Statistical Source Data.

Source Data Fig. 6 Statistical Source Data.

Source Data Fig. 7 Statistical Source Data.

Source Data Extended Data Fig. 3 Statistical Source Data.

Source Data Extended Data Fig. 4 Statistical Source Data.

Source Data Extended Data Fig. 5 Statistical Source Data.

Source Data Extended Data Fig. 6 Statistical Source Data.

Source Data Extended Data Fig. 7 Statistical Source Data.

Source Data Extended Data Fig. 8 Statistical Source Data.

Source Data Extended Data Fig. 8 Unprocessed western gels.

Source Data Extended Data Fig. 9 Statistical Source Data.

## Data Availability

Individual-level patient data are accessible with the consent of the Data Management Committee from the institutions and are not publicly available. The Data Management Committee will review all requests and grant access (if successful). A formal data transfer agreement will be required upon approval. Generally, all requests for access to data will be responded to within 1 month. All data shared will be de-identified. Raw metagenomic sequencing data have been deposited in the NCBI Sequence Read Archive under accession ID PRJNA414688 and are publicly available as of the date of publication. Any additional information required to reanalyse the data reported in this paper is available from the lead contact W.J. (wpjia@sjtu.edu.cn) upon request. Source data are provided with this paper.

## Code availability

No custom code was used in this paper. All publicly available codes and tools used to analyse the data are reported and referenced in the Methods.
