# Supplementary material for: Resistant starch intake facilitates weight loss in humans by reshaping the gut microbiota
Source: Nat Metab. 2024 Feb 26;6(3):578–97. doi: 10.1038/s42255-024-00988-y (PMC10963277; doi:10.1038/s42255-024-00988-y)
Supplement: Supplementary file 1 — Study protocol and statistical analysis plan. [file 42255_2024_988_MOESM1_ESM.pdf]

nature metabolism

Article https://doi.org/10.1038/s42255-024-00988-y

Resistant starch intake facilitates weight loss in humans by reshaping the gut microbiota

In the format provided by the authors and unedited

Supplementary Information

Contents

Study protocol 2

Statistical analysis plan 17

An interventional study of resistant starch on normal-weight and overweight/obese subjects
(A randomized, cross-over trial)

STUDY PROTOCOL

Shanghai Jiao Tong University Affiliated Sixth People's Hospital
Shanghai Key Laboratory of Diabetes Mellitus
Shanghai Clinical Center of Diabetes
Shanghai Diabetes Institute

Version submitted to ChiCTR on July 3rd, 2013

Index

1. Study background and significance……………………………………4
2. Specific aims…………………………………………………………4
3. Research design and methods…………………………………………..4
3.1. overall designs…………………………………………………...4
3.2 statistical power and sample size………………………………...6
4. Inclusion and exclusion criteria……………………………………...7
5. Recruitment…………………………………………………………..7
6. Randomization and blinding…………………………………………7
7. Intervention…………………………………………………………..8
8. Study outcomes………………………………………………………8
9. Participant termination and retention………………………………...8
10. Data collection………………………………………………………9
11. Baseline and follow-up visits………………………………………...11
12. Safety and adverse events…………………………………………...13
13. Drop-outs……………………………………………………………14
14. Ethical requirements…………………………………………………14
15. Data management……………………………………………………14
16. Data analysis plan……………………………………………………14
17. Training for study staff………………………………………………15
18. Reference……………………………………………………………15

1. Study background and significance

With the current global epidemics of obesity, researchers have focused on finding novel ways to prevent weight gain or reduce body weight. This goal is imperative as obesity is a major contributor to chronic metabolic diseases such as diabetes mellitus and cardiovascular disease, which are the leading causes of death worldwide. Conversely, weight loss can ameliorate the related comorbidities, indicating that weight management is crucial for the prevention and treatment of these diseases[1].

Resistant starch (RS) is defined as a kind of prebiotics and dietary fibers that cannot be digested by human amylases in the small intestine and passes into the colon to be fermented by the microbiota. Rodent studies show reduced total body fat, especially visceral fat, in response to RS compared with feeding with digestible starch[2]. However, clinical studies showed that 4 to 12 weeks of RS feeding had no effect on total body weight in subjects with metabolic syndrome[3-5]. RS interventions can reduce the body weight and body fat of rats fed a normal diet, but adding RS to a high-fat diet weakens the intervention effect, indicating that high-fat-diet-induced changes in the gut microbiota decrease RS fermentation and result in reduced effects of RS on adiposity[6]. Thus, the lack of strict diet control may be the reason that RS did not show a significant effect on body weight in the above-mentioned clinical trials. These results also suggests that RS-related gut microbiota plays a crucial role in mediating the therapeutic effects of RS. To date, whether RS can be used as a functional and adaptable food ingredient for treating obesity in humans is unclear, as is how RS-related gut microbiome changes affect adiposity. Therefore, a well-controlled study is needed to explore the influence of RS on the gut microbial community and microbial biotransformation and their relationship to the host's metabolism. To address this issue, we propose to conduct a randomized, crossover-designed clinical trial in normal-weight, overweight and obese individuals on identical diets with moderate fat content, followed by comprehensive metagenomics and metabolomics analysis to investigate safety of RS supplementation, the effect of RS on metabolic phenotype and its influence on gut microbiota and metabolites.

2. Specific aims

We propose to conduct a randomized, double-blind, crossover, and placebo-controlled clinical trial in both normal weight and overweight or obese subjects. There are two sub-studies in this trial. The study subjects of sub-study I will be individuals with normal weight. The safety of RS supplementation will first evaluated in sub-study I. The study subjects of sub-study II will be overweight or obese individuals. Both sub-studies will investigate the effect of RS on metabolic phenotype and its influence on gut microbiota and metabolites.

3. Research design and methods

3.1 Overall designs

3.1.1 Sub-study I: Normal-weight group

This study is a randomized, double-blind, crossover, and placebo-controlled clinical trial. After screening, all eligible subjects will enter a 1-week run-in period and then be randomly assigned into two groups, receiving the RS intervention or control starch (CS). To ensure all subjects have the same dietary intake in the entire study from the run-in period to the experimental period, we will provide them with identical three meals a day, which are designed by dietitians from the Department of Clinical Nutrition, Shanghai Jiao Tong University Affiliated Sixth People's Hospital. The diet is designed according to the Dietary Guidelines for Chinese Residents, with total energy meeting light physical activity needs and containing 50-60% carbohydrate, 25-30% fat, and 15-20% protein. Participants will be asked to receive meal at our institute. They will be allowed one piece of fruit per day, with no extra sugary beverages or snacks recommended. In the crossover study, subjects will receive either resistant starch derived from maize (HAM-RS2, Hi-Maize 260 resistant starch, 22000B00, provided by

Ingredion Inc., Bridgewater, NJ) at 255.4 kcal/day (2.8 kcal/g, 91.2 g, containing 40 g RS) or energy-matched control starch (AMIOCA cornstarch, 04400110, also provided by Ingredion Inc.) at 255.6 kcal/per day (3.55 kcal/g, 72 g, amylopectin, containing 0 g RS) alternately for 4 weeks and separated by a 4-week washout period. The starch will be supplied as powder in ready-to-use sachets to mix with 300 ml water. Participants will take one sachet twice a day, 10 to 15 minutes before meals. Participants and investigators will be unaware of the contents of the study starch and the randomization scheme.

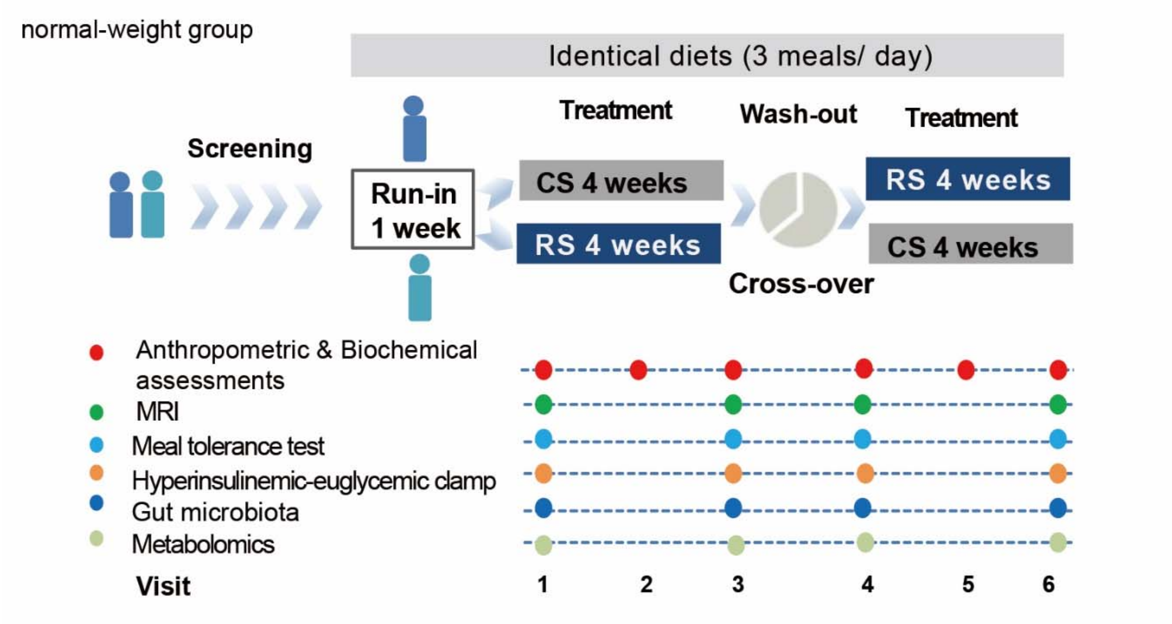

3.1.2 Sub-study II: Overweight/obese group

This study is a randomized, double-blind, crossover, and placebo-controlled clinical trial. After screening, all eligible subjects will enter a 1-week run-in period and then be randomly assigned to two groups, receiving the cross intervention of RS or CS. In the crossover study, subjects will receive either HAM-RS2 (Ingredion Inc., Bridgewater, NJ) at 255.4 kcal/day (2.8 kcal/g, 91.2 g, containing 40 g RS) or energy matched CS (Ingredion Inc.) at 255.6 kcal/per day (3.55 kcal/g, 72 g, amylopectin, containing 0 g RS) alternately for 8 weeks, separated by a 4-week washout period. The starch will be supplied as powder in ready-to-use sachets to mix with 300 ml water. Participants will take one sachet twice a day, 10 to 15 minutes before meals. To ensure all subjects have the same dietary intake in the entire study from the run-in period to the intervention period, we will provide them with identical three meals a day, which are designed by the dietitian from the Department of Clinical Nutrition, Shanghai Jiao Tong University Affiliated Sixth People's Hospital. The diets are designed according to the guidelines for prevention and control of overweight and obesity in Chinese adults[7] and American Heart Association (AHA) / the American College of Cardiology (ACC) / the Obesity Society (TOS) Guideline for the Management of Overweight and Obesity in Adults[8]. The total energy of the diet is 25 kcal multiplied by ideal body weight (kg) [ideal body weight = height (cm) -105 (cm)] per day. The diets contain 50-60% carbohydrate, 25-30% fat, and 15-20% protein. Participants will be asked to receive meals at our institute. Participants will be allowed to eat one piece of fruit per day, with no extra sugary beverages or snacks recommended. Three consecutive 24-hour dietary records (2 weekdays and 1 weekend day) at baseline and the beginning and the end of each intervention period will be collected. The diet information will be collected and daily intake of total energy, macronutrients and certain food components will be calculated using a nutrition treatment computing system (NCCW, Qingdao, China).

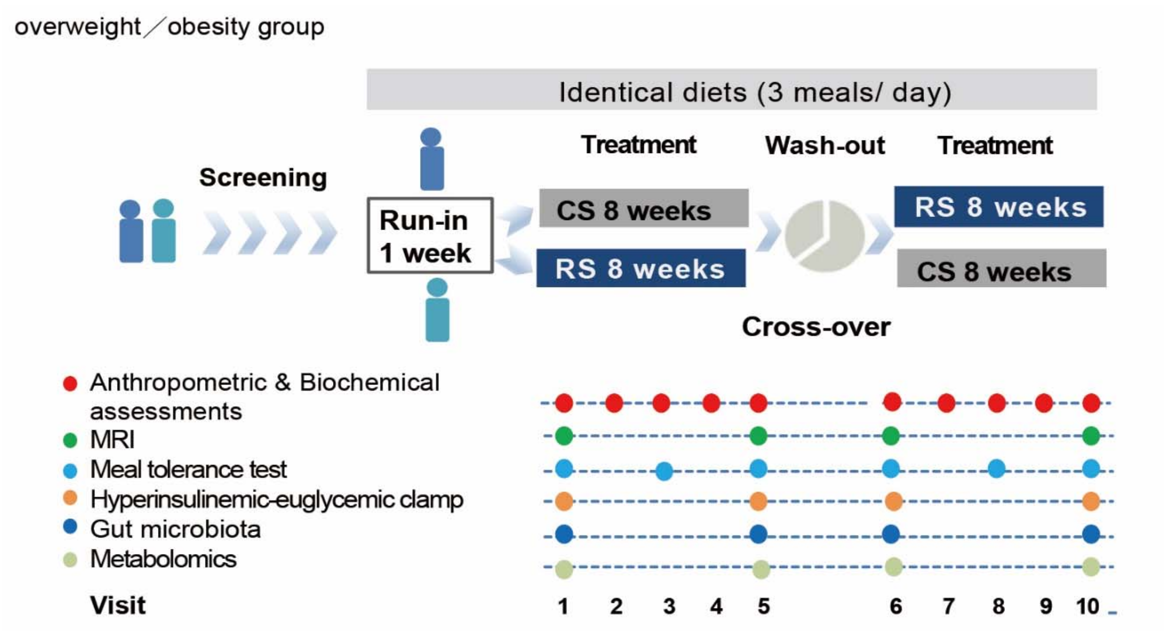

3.1.3 Dietary compliance

Participants will record a study diary for three consecutive 24-hour periods with dietary consumption (amount and type) (2 weekdays and 1 weekend day) at baseline and the beginning and the end of each intervention period including deviations from the dietary instructions. The study diary will also be used for noting any illness and use of antibiotics during the interventions. During each visit, dietary questionnaires will be completed by a trained dietician focusing on consumption of study dietary products and adherence to the dietary regimens in general. We will provide the participants with three meals a day and participants will be asked to receive meals at our institute. A researcher will record when meals are not taken at our institute, which will be another measurement of diet compliance.

3.2 Statistical power and sample size

3.2.1 Sub-study I: Normal-weight group

According to the literature and preliminary studies, we estimate the change in visceral fat area (VFA) and its standard deviation as 3 ± 3cm^2 after the RS intervention. Using the formula $n = \frac{2(u_\alpha + u_{\beta(1)})^2 \sigma^2}{\delta^2}$, with a two-tailed test significance level of 0.05 and a statistical power of 80%, the calculated minimum sample size is 16. To allow for a 17% dropout after randomization, a total of 19 participants will be invited for participation.

3.2.2 Sub-study II: Overweight/obese group

According to the literature and preliminary studies, we estimate the change value in body weight and its standard deviation as 2 ± 2.8 kg after the RS intervention. Using the formula $n = \frac{2(u_\alpha + u_{\beta(1)})^2 \sigma^2}{\delta^2}$, with a two-tailed test significance level of 0.05 and a statistical power of 80%, the calculated minimum sample size is 31. To allow for a 17% dropout after randomization, a total of 37 participants will be invited for participation.

4. Inclusion and Exclusion Criteria

4.1 Sub-study I: Normal-weight group

4.1.1 Inclusion criteria

- Provide oral and written informed consent;
- Chinese;
- 18-55 years old;
- 18.5 kg/m^2 < Body mass index (BMI) < 24 kg/m^2;
- Waist circumference < 85 cm for men or < 80 cm for women.

4.1.2 Exclusion criteria

- Diagnosed with impaired glucose tolerance or diabetes mellitus;
- Chronic disease such as hyperthyroidism, hypothyroidism, gastrointestinal disease, cardio-cerebrovascular disease;
- Use of antibiotics or probiotic supplements within 3 weeks before the study or during the study;
- Regularly use of prescription drugs (not including contraceptives);
- Participation in other clinical trials within the past 4 weeks.

4.2 Sub-study II: Overweight/obese group

4.2.1 Inclusion criteria

- Provide oral and written informed consent;
- Chinese;
- 18-55 years old;
- Overweight or obesity: BMI ⩾ 24 kg/m^2 or waist circumference ⩾ 85 cm for men and ⩾ 80 cm for women.

4.2.2 Exclusion criteria

- Unwilling to accept randomization;
- Presenting with acute diseases or use of antibiotics within the past 3 weeks;
- Chronic disease such as hyperthyroidism, hypothyroidism, diabetes, gastrointestinal disease, cardio-cerebrovascular disease;
- Current treatment with systemic corticosteroids or medications which may affect glucose metabolism;
- Use of antibiotics or probiotic supplements within 3 weeks before the study or during the study;
- Regularly use of prescription drugs (not including contraceptives);
- Participation in other clinical trials within the past 4 weeks.

5. Recruitment

Participants will be recruited by public advertisements from Shanghai, China, from July 2013 to 2016. Once eligibility is confirmed, a physician will fully inform the participant about the experiment. The participant will sign an informed consent, and schedule a baseline visit.

6. Randomization and blinding

6.1 Randomization

Randomization will be conducted by an investigator without contact with the participants. Participants will be randomly assigned to the CS-washout-RS or RS-washout-CS intervention scheme with an allocation ratio of 1:1. Prior to randomization, the study coordinator should confirm that all screening procedures have been completed, and the participant meets all eligibility criteria. The randomization schedule will be generated using SAS PROC PLAN in SAS software.

6.2 Blinding

RS and CS will be packaged in sealed bags that are identical in appearance, and the participants and investigators will be unaware of the contents of the study starch and the randomization scheme during the double-blind period. Only the research designer will know the randomization scheme. Participants, investigators, clinical staff and outcome assessors will be blinded to the allocation sequence. The blinding will be lifted when the bioinformatics analysis will be conducted to explore the potential mechanism by which the gut microbiota confers the physiological benefits of RS.

7. Intervention

7.1 Sub-study I: Normal-weight group

In the crossover study, participants will receive either 255.4 kcal/per day HAM-RS2 (2.8 kcal/g, 91.2 g, containing 40 g RS) or energy matched 255.6 kcal/per day CS (3.55 kcal/g, 72 g, amylopectin, containing 0 g RS) alternately for 4 weeks and separated by a 4-week washout period. During the run-in period and the whole study period, participants will be offered with identical meals.

7.2 Sub-study II: Overweight/obese group

In the crossover study, participants will receive either 255.4 kcal/per day HAM-RS2 (2.8 kcal/g, 91.2 g, containing 40 g RS) or energy matched 255.6 kcal/per day CS (3.55 kcal/g, 72 g, amylopectin, containing 0 g RS) alternately for 8 weeks and separated by a 4-week washout period. During the run-in period and the whole study period, participants will be offered with identical meals.

8. Study Outcomes

8.1 Sub-study I: Normal-weight group

8.1.1 Primary outcome

- VFA

8.1.2 Secondary outcome

(1) Subcutaneous fat area (SFA)
(2) Body weight;
(3) Body fat;
(4) Waist circumference;
(5) Lipid profiles;
(6) Insulin sensitivity;
(7) Metabolome;
(8) Gut microbiome.

8.2 Sub-study II: Overweight/obese group

8.2.1 Primary outcome

- Body weight

8.2.2 Secondary outcome

(1) VFA;
(2) SFA;
(3) Body fat;
(4) Waist circumference;
(5) Lipid profiles;
(6) Insulin sensitivity;
(7) Metabolome;
(8) Gut microbiome.

9. Participant termination and retention

9.1 Participant Termination

- Adverse events or other unexpected reasons;
- Inability to complete the study as required.

9.2 Participants Compliance

- Visits will be scheduled at the convenience of the patients;
- Personalized birthday, holiday, and anniversary cards will be sent to participants. Small gifts will be used to improve participants' connection to the study and research staff;
- Starch will be given every 2 weeks, recycling the empty bags as well as unused starch;
- Family members will be mobilized to support participants;
- Peer education encouraged participants supporting each other.

10. Data collection

10.1 Measurements

- Dietary questionnaire and gastrointestinal adverse reaction questionnaire;
- Recent medications;
- Anthropometric measurements: height, weight, body fat, waist circumference, hip circumference, and blood pressure;
- Glucometabolic and biochemical tests: hyperinsulinemic-euglycemic clamp, meal tolerance test (MTT), lipid profiles, liver and renal functions, blood and urine routine examination;
- Abdominal fat area determined by Magnetic Resonance Imaging (MRI);
- Serum and fecal sample collection.

10.2 Methods

10.2.1 Anthropometric measurements

After an overnight fasting for at least 10 hours, participants will come to Shanghai Diabetes Mellitus Clinical Study Center at 7:30 a.m. to 8:30 a.m. Participants will be measured in light clothing without shoes, hat or any ornament.

- Height: Both feet will naturally placed on the base plate of a measuring ruler, participants will be asked to straighten their body and legs with heels, back, and head close to the measuring ruler. Upper limbs will be positioned naturally. During measurements, the hair of the participants will be pressed flat. Measured values are in cm.
- Body weight and body fat percentage: Participants will be measured with a body composition analyzer (Tanita, TBF-410, Tokyo, Japan), calibrating the accuracy and sensitivity of the meter before the measurements. Participants should empty bladder, and remove clothing, shoes, and socks, and wear light clothing before testing. Participants should keep their hands and feet dry and stand quietly in the center of the analyzer, with their feet in full contact with an iron plate, and holding an iron plate with both hands. The report sheet will output a moment after the reading of weight meter is stable.
- Waist circumference: Waist circumference will be measured at the midpoint between the inferior costal margin and the superior border of the iliac crest on the midaxillary line.
- Hip circumstance: Hip circumstance will be measured at the widest part over the greater trochanters by the same researcher. The measured values are in cm.
- Blood pressure: Participants should avoid strenuous exercise, and will be seated in a quiet environment with appropriate temperature for 5-10 minutes before measurements. When measuring blood pressure, the arm (usually the right upper arm) to be measured needs to be exposed and at the same level as the heart. The cuff of a sphygmomanometer should be placed close to the brachial artery of the elbow socket, and the lower edge of the cuff

should be 3-4 cm away from the elbow socket. The reading on the convex surface of a mercury column at the first phase of a Korotkoff sound is a systolic reading, and the reading on the convex surface of a mercury column at the fifth phase of a Korotkoff sound is a diastolic reading. Blood pressure measurements will be taken three times at least 2 minutes apart and then averaged.

10.2.2 Oral glucose tolerance test (OGTT)
Participants will fast from 8 p.m. the day before the test. On the day of the test, after a fasting blood sample is drawn, participants will drink 75g glucose in 250-300 ml water within 5 minutes. Blood samples will be drawn at 30min, 60min, 90min, 120min, and 180min after glucose loading to measure glucose, insulin, and C-peptide.

10.2.3 MTT
Participants will fast from 8 p.m. the day before the test. On the day of the test, after a fasting blood sample is drawn, participants will take a standard meal (80g non-fried instant noodle without soup: 315.2 kcal including 68.4 g carbohydrate and 10.4 g protein) within 5 minutes. Blood samples will be drawn respectively at 30min, 60min, 90min, 120min, and 180min after the meal to measure glucose, insulin, and C-peptide concentrations.

10.2.4 Hyperinsulinemic-euglycemic clamp
Participants will fast from 8 p.m. the day before the test. Before 8 a.m. of the test day, participants will rest in a supine position after urination and indwelling needles will be placed in veins of both arms. One is used for venous blood collection, and the other is for insulin and glucose infusion. After taking basic blood samples at 30 min, 15 min, and 0 min before the experiment, place the forearm in a thermostat (70% humidity and temperature 50 ^{o}C) to ensure arterialization of the venous blood sample. We will use 0.9% saline to maintain venous access on the blood collection side. The other vein will start to infuse insulin and 20% glucose simultaneously. We will infuse short-acting human insulin solution (40 U/ml) within 10 minutes after the clamping to rapidly increased insulin levels. Insulin will then continuously administered at a rate of 40mU/m^2•min for the following 140 minutes. During the clamp, arterialized venous blood will be taken every 5 minutes to measure blood glucose, C-peptide, and insulin within 0-30 minutes. Blood will be taken every 5 minutes to measure blood glucose, and every 10 minutes to measure C-peptide and insulin for the rest of the experiment. During the infusion, the infusion rate of 20% glucose solution will be adjusted according to the empirical value of DeFronzo's body surface area and the blood glucose level measured during the experiment, so that the clamp blood glucose will be maintained at the basic level of about 5mmol/L. Adjustment time will be recorded. The variation coefficient in blood glucose during the hyperinsulinemic-euglycemic clamp should be less than 5%. All serum samples will be stored at -80 ℃ after centrifuge. All urine samples during the test will be collected to determine the content of urine sugar, which is used to calibrate the clearance rate of glucose[9,10].

10.2.5 MRI
Visceral and subcutaneous adipose tissue areas will be assessed using a 3.0 T clinical MRI scanner (Archiva, Philips Medical System, Amsterdam, The Netherlands). MRI scans will be performed by experienced radiologists using standard array coils that are parallel to the abdomen between the L4 and L5 vertebrae in the supine position. Images will be segmented and calculated for VFA and SFA using SliceOmatic image analysis software (Tomovision Inc., Montreal, QC, Canada) by two trained investigators.

10.2.6 Gastrointestinal symptoms evaluation
After each treatment in this study, participants will be given questionnaires to record the

incidence and extent of defecation and gastrointestinal symptoms after consuming study starch, including nausea, audible bowel sounds, abdominal pain, bloating, and flatulence, as well as consistency in defecation frequency and stool (normal, hard, or watery).

10.2.7 Blood glucose, C-peptide, insulin, and biochemical assessments
Serum samples will be stored at -80℃ before analysis. Fasting plasma glucose and postprandial plasma glucose will be measured by standard glucose oxidase method.

10.2.8 Metabonomic analysis of human serum and urine samples
Metabonomic analysis of human serum and urine samples will be measured by ultra-performance liquid chromatography coupled to a mass spectrometer.

10.2.9 Quantitative detection of SCFAs in human serum and fecal samples
Serum and fecal SCFAs will be measured using gas chromatography.

10.2.10 Fecal sample collection and DNA extraction
Fecal samples will be collected using a tube with DNA stabilizer and stored at -80℃ before analysis. Stool DNA will be extracted.

10.2.11 16S rRNA sequencing (Sub-study I: normal-weight group)

10.2.12 Construction and sequencing of metagenomic library (Sub-study II: overweight/obese group)

11. Baseline and Follow-up Visits
11.1 Screening visit
1. Past history: diet habits, alcohol and cigarette habits, past medical history, medication, weight fluctuation history;
2. Anthropometric measurements: height, weight, body fat, waist circumference, hip circumference, and blood pressure;
3. Glucometabolic and biochemical measurements: OGTT, lipid profile, liver and renal function examination, blood and urine routine examinations.

11.2 Follow-up Visits
11.2.1 Sub-study I: Normal-weight group
11.2.1.1 Visit 1 (0-week)
1. Diet questionnaire and gastrointestinal adverse reaction questionnaire;
2. Recent medication use;
3. Anthropometric measurements: height, weight, body fat, waist circumference, hip circumference, and blood pressure;
4. Glucometabolic and biochemical measurements: hyperinsulinemic-euglycemic clamp, MTT, lipid profile, liver and renal function examination, blood and urine routine examinations;
5. Abdominal fat area by MRI;
6. Serum and fecal samples collection.

11.2.1.2 Visit 2 (2-week±2 days)
1. Diet questionnaire and gastrointestinal adverse reaction questionnaire;
2. Recent medication use;
3. Anthropometric measurements: height, weight, body fat, waist circumference, hip circumference, and blood pressure;
4. Glucometabolic and biochemical measurements:fasting blood glucose and insulin

examination, lipid profile, liver and renal function examination;
5. Serum and fecal samples collection.

11.2.1.3 Visit 3 (4-week±2 days)
1. Diet questionnaire and gastrointestinal adverse reaction questionnaire;
2. Recent medication use;
3. Anthropometric measurements: height, weight, body fat, waist circumference, hip circumference, and blood pressure;
4. Glucometabolic and biochemical measurements: hyperinsulinemic-euglycemic clamp, MTT, lipid profile, liver, and renal function examination;
5. Abdominal fat area by MRI;
6. Serum and fecal samples collection.
11.2.1.4 Visit 4 (8-week±2 days): same as Visit 1
11.2.1.5 Visit 5 (10-week±2 days): same as Visit 2
11.2.1.6 Visit 6 (12-week±2 days): same as Visit 3

11.2.2 Sub-study II: Overweight/obese group
11.2.2.1 Visit 1 (0-week)
1. Diet questionnaire and gastrointestinal adverse reaction questionnaire;
2. Recent medication use;
3. Anthropometric measurements: height, weight, body fat, waist circumference, hip circumference, and blood pressure;
4. Glucometabolic and biochemical measurements: hyperinsulinemic-euglycemic clamp, MTT, lipid profile, liver and renal function examination, blood and urine routine;
5. Abdominal fat area by MRI;
6. Serum and fecal samples collection.

11.2.2.2 Visit 2 (2-week±2 days)
1. Diet questionnaire and gastrointestinal adverse reaction questionnaire;
2. Recent medication use;
3. Anthropometric measurements: height, weight, body fat, Waist circumference, hip circumference, and blood pressure;
4. Glucometabolic and biochemical measurements: fasting blood glucose and insulin examination, lipid profile, liver and renal function examination;
5. Serum and fecal samples collection.

11.2.2.3 Visit 3 (4-week±2 days)
1. Diet questionnaire and gastrointestinal adverse reaction questionnaire;
2. Recent medication use;
3. Anthropometric measurements: height, weight, body fat, waist circumference, hip circumference, and blood pressure;
4. Glucometabolic and biochemical measurements: MTT, lipid profile, liver and renal function examination;
5. Serum and fecal samples collection.

11.2.2.4 Visit 4 (6-week±2 days): same as Visit 2

11.2.2.5 Visit 5 (8-week±2 days)
1. Diet questionnaire and gastrointestinal adverse reaction questionnaire;
2. Recent medication use;
3. Anthropometric measurements: height, weight, body fat, waist circumference, hip

circumference, and blood pressure;
4. Glucometabolic and biochemical measurements: hyperinsulinemic-euglycemic clamp, MTT, lipid profile, liver and renal function examination;
5. Abdominal fat area by MRI;
6. Serum and fecal samples collection.

11.2.2.6 Visit 6 (12-week±2 days): same as Visit 1
11.2.2.7 Visit 7 (14-week±2 days): same as Visit 2
11.2.2.8 Visit 8 (16-week±2 days): same as Visit 3
11.2.2.9 Visit 9 (18-week±2 days): same as Visit 2
11.2.2.10 Visit 10 (20-week±2 days): same as Visit 5

12. Safety and adverse events

12.1 Safety

During the study, participants' vital signs and laboratory examinations will be observed. All participants will be evaluated for their pulse rate and blood pressure and will be evaluated for any physical discomfort, including gastrointestinal adverse reaction or other adverse events.

12.2 Adverse events (AEs)

AEs are defined as any undesirable experience occurring to a participant during the study, whether or not they are considered related to the experimental intervention. Adverse medical conditions could be symptoms (for example, nausea, chest pain), physical signs (for example, tachycardias, hepatomegaly), or abnormal examination results (for example, laboratory examinations, electrocardiogram). In this clinical study, adverse events include adverse medical events happening at any time, even in the screening period before any study treatment.

12.3 Serious adverse events (SAEs)

SAEs are defined as any untoward medical occurrence or effect that resulted in death; is life-threatening (at the time of the event); requires hospitalization or prolongation of existing inpatients' hospitalization; results in persistent or significant disability or incapacity; or any other important medical event that does not result in any of the outcomes listed above.

12.4 Recording of adverse events

- Participants should be informed and required to report dynamic disease changes. Physicians should avoid induced questions. Adverse events or unexpected side effects (including symptoms, physical signs, and laboratory examinations) will be under observation during the study. Additionally, physicians will analyze the causes, make a judgment, keep track of and record the incidences of adverse reactions and adverse events.
- For adverse events occurring during the study, their nature, severity, level, duration, treatment, and prognosis will be recorded on CRF. Physicians will evaluate the causal-effect relationship and record with signature and date.

12.5 Reporting of severe adverse events

Physicians will immediately provide appropriate care when severe adverse events occur in participants, to ensure their safety. Additionally, physicians should report the adverse events and treatment to the Principal Investigator Prof. Jia Weiping and the Human Research Ethics Committee of the Shanghai Sixth People's Hospital within 24 hours.

12.6 Treatment of Serious adverse events

Once severe adverse events occur, physicians will decide whether to suspend study or not

according to the condition. All adverse events will be followed and recorded, including the progress and results of the treatment.

12.7 Follow-up visit, time and prognosis of serious adverse events

- Follow-up visit will include outpatient service, home visit, telephone and messages.
- Follow-up visit time and prognosis will depend on severity and degree. For mild adverse events, participants should be followed until the adverse events have completely subsided; for serious adverse events, participants should be kept following after the adverse events completely subsided, according to condition.

13. Dropouts

Participants could choose to discontinue the use of study starch or withdraw from the study voluntarily. Participants who drop out of the trial will be required to return to the outpatient service for a site termination visit. Physicians will record the reason for dropping out and any adverse events in a case report form. We will continue to observe or evaluate participants who withdraw and follow up on adverse events, if possible. Participants who dropped out should return all study materials.

14. Ethical requirements

The study was approved by the Human Research Ethics Committee of the Shanghai Sixth People's Hospital, following the principle of the declaration of Helsinki. Written informed consent will be obtained from all participants.

15. Data management

15.1 All CRF for each participant should be filled out by study staff. The CRF is used to collect information during the study. Original files will be kept intact, stored in a fixed place, and locked. According to Chinese GCP principles, files should be kept for at least 5 years.
15.2 The data recorded in CRF by physicians will be the raw data of this study.
15.3 All data will be double-entered by researcher staff.
15.4 After establishing and regarding as correct in blind review, the database will be locked, and no alteration can be made.

16. Data analysis plan (more details described in statistical analysis protocol [SAP])
16.1 Study outcomes
16.1.1 Sub-study I: Normal-weight group

- The primary study outcome is change of VFA after RS intervention vs. CS intervention.
- The secondary outcomes are (1) SFA; (2) body fat; (3) waist circumference; (4) body weight; (5) lipid profiles; (6) insulin sensitivity; (7) metabolome and (8) gut microbiome.

16.1.2 Sub-study II: Overweight/obese group

- The primary study outcome is change of body weight after RS intervention vs. CS intervention.
- The secondary outcomes are (1) VFA; (2) SFA; (3) body fat; (4) waist circumference; (5) lipid profiles; (6) insulin sensitivity; (7) metabolome; and (8) gut microbiome.

16.2 Analysis methods
16.2.1 Analytic methods of clinical indicators

All statistical analyses will be made using the SPSS 17.0 software (SPSS, Inc, Chicago, IL). For descriptive analysis, normally distributed data will be shown as mean ± sd, and data that are not normally distributed, as determined using the Kolmogorov-Smirnov test, will be expressed as median with interquartile range. Two-sided p values <0.05 are considered significant. The effects of the interventions on all outcomes will be analyzed by the linear

mixed model or generalized estimating equation adjusted for age, gender and carry-over effect. Bonferroni adjustment will be used as the method of the multiple testing correction.

16.2.2 Bioinformatics analysis of 16S rRNA sequencing data
Statistical comparisons of microbiota alpha and beta diversity will be performed with Wilcoxon signed-rank test (or Wilcoxon rank-sum test) and Permutational multivariate analysis of variance, respectively. The relative abundances in each sample at various taxonomic levels will be calculated and statistically compared before and after the 4-week RS or CS treatments in normal-weight subjects using the Wilcoxon signed-rank test. Correlation analysis between microbial taxa (genera or species) and phenotypes or metabolites will be performed using Spearman rank-based correlations or partial Spearman rank-based correlations when there is a need to adjust for cofounding factors.

16.2.3 Bioinformatics analysis of shotgun metagenomic data
Statistical comparisons of microbiota alpha and beta diversity will be performed with Wilcoxon signed-rank test (or Wilcoxon rank-sum test) and Permutational multivariate analysis of variance, respectively. The relative abundances in each sample at various taxonomic levels will be calculated and statistically compared before and after the 8-week RS or CS treatments in overweight or obese subjects using the Wilcoxon signed-rank test. Correlation analysis between microbial taxa (genera or species) and phenotypes or metabolites will be performed using Spearman rank-based correlations or partial Spearman rank-based correlations when there is a need to adjust for cofounding factors. Linear regression analysis using the phenotypes variation as the dependent variable and the species abundance variation as the independent variable will be carried out.

17. Training for study staff
1) All study staff (physicians and nurses) will be trained and certified prior to the start of the study to familiarize themselves with the methods and procedures of this trial.
2) Physicians should ensure all participants are able to understand the significance, benefit, and risk of this trial, and ensure that they comply with the treatment and observation as the trial required.
3) Physicians should record the CRF faithfully and detailedly. All data will be double-entered by researcher staff.
4) Clinical trial operation and sample collection should be conducted by appointed physicians, and laboratory examinations should be conducted by appointed lab technicians.
5) The report of examinations must be complete, including date, testing items, results and its normal range.

18. Reference

1. Margetts, B. WHO global strategy on diet, physical activity and health.Editorial. *Public health nutrition* **7**, 361-363 (2004).
2. Pawlak, D.B., Kushner, J.A. & Ludwig, D.S. Effects of dietary glycaemic index on adiposity, glucose homoeostasis, and plasma lipids in animals. *Lancet* **364**, 778-785 (2004).
3. Robertson, M.D.*, et al.* Insulin-sensitizing effects on muscle and adipose tissue after dietary fiber intake in men and women with metabolic syndrome. *J Clin Endocrinol Metab* **97**, 3326-3332 (2012).
4. Johnston, K.L., Thomas, E.L., Bell, J.D., Frost, G.S. & Robertson, M.D. Resistant starch improves insulin sensitivity in metabolic syndrome. *Diabetic medicine : a journal of the British Diabetic Association* **27**, 391-397 (2010).
5. Maki, K.C.*, et al.* Resistant starch from high-amylose maize increases insulin sensitivity in overweight and obese men. *J Nutr* **142**, 717-723 (2012).
6. Charrier, J.A.*, et al.* High fat diet partially attenuates fermentation responses in rats fed resistant starch from high-amylose maize. *Obesity (Silver Spring, Md.)* **21**, 2350-2355 (2013).
7. Chen, C., Lu, F.C. & Department of Disease Control Ministry of Health, P.R.C. The guidelines for prevention and control of overweight and obesity in Chinese adults. *Biomed Environ Sci* **17 Suppl**, 1-36

(2004).
8. Jensen, M.D., *et al.* 2013 AHA/ACC/TOS guideline for the management of overweight and obesity in adults: a report of the American College of Cardiology/American Heart Association Task Force on Practice Guidelines and The Obesity Society. *Circulation* **129**, S102-138 (2014).
9. DeFronzo, R.A., Tobin, J.D. & Andres, R. Glucose clamp technique: a method for quantifying insulin secretion and resistance. *Am J Physiol* **237**, E214-223 (1979).
10. Jia, W., *et al.* Association of serum retinol-binding protein 4 and visceral adiposity in Chinese subjects with and without type 2 diabetes. *The Journal of clinical endocrinology and metabolism* **92**, 3224-3229 (2007).

PROTOCOL
An interventional study of resistant starch on normal-weight and overweight/obese subjects
(A randomized, cross-over trial)

STATISTICAL ANALYSIS PLAN

Shanghai Jiao Tong University Affiliated Sixth People's Hospital
Shanghai Key Laboratory of Diabetes Mellitus
Shanghai Clinical Center of Diabetes
Shanghai Diabetes Institute

Version submitted to ChiCTR on July 3, 2013

Index

1. SYNOPSIS OF STUDY DESIGN AND PROCEDURES 19
1.1 Study Objective 19
1.2 Study Design 19
1.2.1 Study Outcome...19
1.2.2 Safety Outcome 19
1.3 Analysis Populations 20
1.3.1 Intent-to-Treat Population..20
1.3.2 Full Analysis Set...20
1.4 Sample Size and Power Calculation 20
2. ANALYSIS CONSIDERATIONS 20
2.1 Demographic and Baseline Characteristics..20
2.2 Endpoint Analyses 20
2.2.1 Effectiveness Endpoint Analyses 20
2.2.2 Safety Outcome Analyses...21
2.3 Subgroup Analysis..21
2.4 Additional Descriptive Analyss.. 21

1. SYNOPSIS OF STUDY DESIGN AND PROCEDURES

The purpose of the statistical analysis plan (SAP) is to provide a detailed and comprehensive description of the planned methodology and analysis for interventional study of resistant starch on normal-weight and overweight/obese subjects. This plan is based on the study protocol dated July 3rd, 2013.

1.1 Study Objective

We propose to conduct a randomized, double-blind, crossover, and placebo-controlled clinical trial in both normal weight and overweight or obese subjects. There are two sub-studies in this trial. The study subjects of sub-study I will be individuals with normal weight. The safety of RS supplementation will first evaluated in sub-study I. The study subjects of sub-study II will be overweight or obese individuals. The primary objective of this study is to obtain safety and effectiveness data on participants. Both sub-studies will investigate the effect of RS on metabolic phenotype and its influence on gut microbiota and metabolites.

1.2 Study Design

This will be a randomized, double-blind, crossover, single center and placebo-controlled study.

1.2.1 Study Outcomes

Sub-study I: Normal-weight group

Primary outcome

- Visceral fat area (VFA)

Secondary outcome

- Subcutaneous fat area (SFA)
- Body weight;
- Body fat;
- Waist circumference;
- Lipid profiles;
- Insulin sensitivity;
- Metabolome;
- Gut microbiome.

Sub-study II: Overweight/obese group

Primary outcome

- Body weight

Secondary outcome

- VFA;
- SFA;
- Body fat;
- Waist circumference ;
- Lipid profiles;
- Insulin sensitivity;
- Metabolome;
- Gut microbiome.

1.2.2 Safety Outcome

During the study, participants' vital signs and laboratory examinations will be observed. All participants will be evaluated for their pulse rate and blood pressure and will be evaluated for any physical discomfort, including gastrointestinal adverse reaction or other adverse events.

Adverse events (AEs) are defined as any undesirable experience occurring to a participant during the study, whether or not they are considered related to the experimental intervention. Adverse medical conditions could be symptoms (for example, nausea, chest pain), physical

signs (for example, tachycardias, hepatomegaly), or abnormal examination results (for example, laboratory examinations, electrocardiogram). In this clinical study, adverse events include adverse medical events happening at any time, even in the screening period before any study treatment.

Serious adverse events (SAEs) are defined as any untoward medical occurrence or effect that resulted in death; is life-threatening (at the time of the event); requires hospitalization or prolongation of existing inpatients' hospitalization; results in persistent or significant disability or incapacity; or any other important medical event that does not result in any of the outcomes listed above.

1.3 Analysis Populations

1.3.1 Intent-to-Treat (ITT) Population

The ITT population will include participants who enrolled in the study, regardless of whether or not treatment was attempted.

1.3.2 Full Analysis Set (FAS)

The FAS will includes all participants randomized to treatment who received at least one dose of the assigned treatment.

1.4 Sample Size and Power Calculation

Sub-study I: Normal-weight group

According to the literature and preliminary studies, we estimate the change in VFA and its standard deviation as $3 \pm 3cm^2$ after the RS intervention. With a two-tailed test significance level of 0.05 and a statistical power of 80%, the calculated minimum sample size is 16. To allow for a 17% dropout after randomization, a total of 19 participants will be invited for participation.

Sub-study II: Overweight/obese group

According to the literature and preliminary studies, we estimate the change value in body weight and its standard deviation as 2 ± 2.8 kg after the RS intervention. With a two-tailed test significance level of 0.05 and a statistical power of 80%, the calculated minimum sample size is 31. To allow for a 17% dropout after randomization, a total of 37 participants will be invited for participation.

2. ANALYSIS CONSIDERATIONS

2.1 Demographic and Baseline Characteristics

Demographic and baseline characteristics will be summarized. Baseline is defined as the last assessment evaluated prior to treatment. Demographic and baseline characteristics will be presented with summary statistics (sample size, mean, standard deviation, median, and interquartile range) for continuous variables and frequency distributions for categorical variables.

2.2 Endpoint Analyses

2.2.1 Effectiveness Endpoint Analyses

2.2.1.1 Analytic methods of clinical indicators

All statistical analyses will be made using the SPSS 17.0 software (SPSS, Inc, Chicago, IL). For descriptive analysis, normally distributed data will be shown as mean ± sd, and data that are not normally distributed, as determined using the Kolmogorov-Smirnov test, will be expressed as median with interquartile range. Two-sided p values <0.05 are considered significant. We will compare the actual changes between the groups (RS vs CS) and the with-in group change using the linear mixed model or generalized estimating equation. The effects

of the interventions on all outcomes will be adjusted for age, gender and carry-over effect. Bonferroni adjustment will be used as the method of the multiple testing correction.

2.2.1.2 Bioinformatics analysis of 16S rRNA sequencing data

Statistical comparisons of microbiota alpha and beta diversity (unweighted and weighted UniFrac distances) will be performed with Wilcoxon signed-rank test (or Wilcoxon rank-sum test) and Permutational multivariate analysis of variance (PERMANOVA), respectively. The relative abundances in each sample at various taxonomic levels will be calculated and statistically compared before and after the 4-week RS or CS treatments in normal-weight subjects using the Wilcoxon signed-rank test via the SPSS 17.0 software (SPSS Inc., Chicago, IL, USA) or R 3.0.0 (or above). Correlation analysis between microbial taxa (genera or species) and phenotypes or metabolites will be performed using Spearman rank-based correlations or partial Spearman rank-based correlations when there is a need to adjust for cofounding factors.

2.2.1.3 Bioinformatics analysis of shotgun metagenomic data

Statistical comparisons of microbiota alpha and beta diversity (unweighted and weighted UniFrac distances) will be performed with Wilcoxon signed-rank test (or Wilcoxon rank-sum test) and Permutational multivariate analysis of variance (PERMANOVA), respectively. The relative abundances in each sample at various taxonomic levels will be calculated and statistically compared before and after the 8-week RS or CS treatments in overweight or obese subjects using the Wilcoxon signed-rank test via the SPSS 17.0 software (SPSS Inc., Chicago, IL, USA) or R 3.0.0 (or above). Correlation analysis between microbial taxa (genera or species) and phenotypes or metabolites will be performed using Spearman rank-based correlations or partial Spearman rank-based correlations when there is a need to adjust for cofounding factors. Linear regression analysis using the phenotypes variation (fold-change after intervention) as the dependent variable and the species abundance variation as the independent variable (features) will be carried out with the R package glmulti. The non-metric multidimensional scaling ordination and correlations calculation between the variation of the species abundance/phenotypes and the overall profiles will be carried out using R package VEGAN.

2.2.2 Safety Outcome Analyses

Overall summaries of AEs and SAEs will be presented.

2.3 Subgroup Analysis

The following subgroup analyses will be presented for effectiveness and safety endpoints.

- Impaired glucose tolerance (IGT) vs. normal glucose tolerance (NGT)

2.4 Additional Descriptive Analysis

Additional descriptive summary statistics will be presented for clinical assessments, procedural and device data, and efficacy results. For continuous variables, summary statistics (sample size, mean, standard deviation, median, and interquartile range). For categorical variables, results will be summarized with frequency distributions.
