# Supplementary material for: Resistant starch intake facilitates weight loss in humans by reshaping the gut microbiota
Source: Nat Metab. 2024 Feb 26;6(3):578–97. doi: 10.1038/s42255-024-00988-y (PMC10963277; doi:10.1038/s42255-024-00988-y)
Supplement: Supplementary file 2 — Reporting Summary [file 42255_2024_988_MOESM2_ESM.pdf]

nature portfolio

Corresponding author(s): Weiping Jia, Aimin Xu, Gianni Panagiotou, Huating Li and Xinyu Liu

Last updated by author(s): Jan 4, 2024

Reporting Summary

Nature Portfolio wishes to improve the reproducibility of the work that we publish. This form provides structure for consistency and transparency in reporting. For further information on Nature Portfolio policies, see our Editorial Policies and the Editorial Policy Checklist.

Statistics

For all statistical analyses, confirm that the following items are present in the figure legend, table legend, main text, or Methods section.

n/a	Confirmed	
☐	☒	The exact sample size (*n*) for each experimental group/condition, given as a discrete number and unit of measurement
☐	☒	A statement on whether measurements were taken from distinct samples or whether the same sample was measured repeatedly
☐	☒	The statistical test(s) used AND whether they are one- or two-sided<br>*Only common tests should be described solely by name; describe more complex techniques in the Methods section.*
☐	☒	A description of all covariates tested
☐	☒	A description of any assumptions or corrections, such as tests of normality and adjustment for multiple comparisons
☐	☒	A full description of the statistical parameters including central tendency (e.g. means) or other basic estimates (e.g. regression coefficient) AND variation (e.g. standard deviation) or associated estimates of uncertainty (e.g. confidence intervals)
☐	☒	For null hypothesis testing, the test statistic (e.g. *F*, *t*, *r*) with confidence intervals, effect sizes, degrees of freedom and *P* value noted<br>*Give P values as exact values whenever suitable.*
☒	☐	For Bayesian analysis, information on the choice of priors and Markov chain Monte Carlo settings
☐	☒	For hierarchical and complex designs, identification of the appropriate level for tests and full reporting of outcomes
☐	☒	Estimates of effect sizes (e.g. Cohen's *d*, Pearson's *r*), indicating how they were calculated

Our web collection on statistics for biologists contains articles on many of the points above.

Software and code

Policy information about availability of computer code

Data collection	EpiData 3.1 was used for data entry and documentation.
Data analysis	SPSS version 17, R 3.3.2 and Image J 1.54f software were used for data analysis.

For manuscripts utilizing custom algorithms or software that are central to the research but not yet described in published literature, software must be made available to editors and reviewers. We strongly encourage code deposition in a community repository (e.g. GitHub). See the Nature Portfolio guidelines for submitting code & software for further information.

Data

Policy information about availability of data

All manuscripts must include a data availability statement. This statement should provide the following information, where applicable:

- Accession codes, unique identifiers, or web links for publicly available datasets
- A description of any restrictions on data availability
- For clinical datasets or third party data, please ensure that the statement adheres to our policy

Individual-level patient data can be accessible with the consent of Data Management Committee from institutions and are not publicly available. The Data Management Committee will then review all the requests and grant (if successful). A formal data transfer agreement will be required upon approval. Generally, all these requests for access to the data will be responded to within 1month. All data shared will be de-identified. Raw metagenomic sequencing data have been

deposited in NCBI Sequence Read Archive under accession ID PRJNA414688 and are publicly available as of the date of publication. Any additional information required to reanalyze the data reported in this paper is available from the lead contact Weiping Jia (wpjia@sjtu.edu.cn) upon request.

Research involving human participants, their data, or biological material

Policy information about studies with human participants or human data. See also policy information about sex, gender (identity/presentation), and sexual orientation and race, ethnicity and racism.

Reporting on sex and gender	There were 37 subjects with overweight or obese who completed the trial, including 22 male and 15 female subjects.
Reporting on race, ethnicity, or other socially relevant groupings	No socially constructed or socially relevant categorization was made in the selection of study participants.
Population characteristics	The average age of the subjects was 33.43±7.71 years (mean ± SD). Subjects with the following conditions were excluded from this study: acute illness or using the antibiotics 3 weeks prior to the study, known hyperthyroidism or hypothyroidism, known diabetes, current treatment with systemic corticosteroids or medications that may affect glucose metabolism, participating or have participated in other clinical trials 4 weeks before the study.
Recruitment	Participants will be recruited by public advertisements from Shanghai, China, from July 2013 to 2016. Once eligibility is confirmed, a physician will fully inform the participant about the experiment. As participants self-referred to the study, this may have caused a selection bias towards healthier persons. Consequently, the study results may not be generalized to the entire population living with excess body weight.
Ethics oversight	The study was approved by the Ethics Committee of Shanghai Jiao Tong University Affiliated Sixth People's Hospital, following the principle of the Declaration of Helsinki.

Note that full information on the approval of the study protocol must also be provided in the manuscript.

Field-specific reporting

Please select the one below that is the best fit for your research. If you are not sure, read the appropriate sections before making your selection.

☒ Life sciences ☐ Behavioural & social sciences ☐ Ecological, evolutionary & environmental sciences

For a reference copy of the document with all sections, see nature.com/documents/nr-reporting-summary-flat.pdf

Life sciences study design

All studies must disclose on these points even when the disclosure is negative.

Sample size	Human study: We estimated the change value of body weight and its standard deviation as 2 ± 2.8 kg after RS intervention. With a two-tailed test significance level of 0.05 and a statistical power of 80%, the calculated minimum sample size was 31. To allow for a 17% dropout after randomization, a total of 37 participants were invited for participation.<br>Animal study: The sample sizes of animal experiments were estimated according to previous studies (PMID: 37872351, PMID: 36646754, PMID: 37365376) and the known variability of the assays.
Data exclusions	No data were excluded from the analysis.
Replication	Human study: N/A due to the nature of an dietary intervention.<br>All the animal experiments were repeated three times independently. All results are reproducible.
Randomization	An independent researcher performed the randomization and assigned participants to a CS-washout-RS or RS-washout-CS intervention scheme with an allocation ration of 1:1. The randomization schedule will be generated using SAS PROC PLAN in SAS software. For all experiments, participants and research animals were randomly allocated into different groups.
Blinding	RS and CS were packaged in sealed bags that were identical in appearance, and participants and investigators were unaware of the contents of the study starch and the randomization scheme during the double-blind period. Only the research designer knew the randomization scheme. Participants, investigators, clinical staff and outcome assessors were blinded to the allocation sequence during data collection and analysis. The blinding was lifted when the bioinformatics analysis were conducted to explore the potential mechanism by which the gut microbiota confers the physiological benefits of RS. The investigators were not blinded to animal experiments as the treatment of each mouse would need to be known to the person handling the mice. When feasible, data analysis was performed blind.

Reporting for specific materials, systems and methods

We require information from authors about some types of materials, experimental systems and methods used in many studies. Here, indicate whether each material, system or method listed is relevant to your study. If you are not sure if a list item applies to your research, read the appropriate section before selecting a response.

Materials & experimental systems

n/a	Involved in the study
☐	☒ Antibodies
☒	☐ Eukaryotic cell lines
☒	☐ Palaeontology and archaeology
☐	☒ Animals and other organisms
☐	☒ Clinical data
☒	☐ Dual use research of concern
☒	☐ Plants

Methods

n/a	Involved in the study
☒	☐ ChIP-seq
☒	☐ Flow cytometry
☒	☐ MRI-based neuroimaging

Antibodies

Antibodies used	ZO1 antibody (Abcam, ab96587, 1:500 dilution, Rabbit polyclonal to ZO1 tight junction protein) ; Occludin antibody (Abcam, ab216327, 1:100 dilution, Rabbit monoclonal [EPR20992] to Occludin); ANGPTL4 antibody (Proteintech, 18374-1-AP, 1: 500 dilution, Rabbit polyclonal to ANGPTL4); Erk1/2 antibody (Cell Signaling Technology, 9102, 1:1000 dilution, Rabbit polyclonal to Erk1/2), p-Erk1/2 (Thr202/Tyr204) (Cell Signaling Technology, 9101, 1:1000 dilution, Rabbit polyclonal to p- Erk1/2); Rabbit IgG (H+L) secondary antibody (Thermo, 31460, 1: 2500 dilution).
Validation	The ZO1 antibody and Occludin antibody were validated in our laboratory (Circulation 133, 2434-2446, 2016). The Erk1/2 and p-Erk1/2 antibody were validated in our laboratory (Front Endocrinol (Lausanne) 30:12:773340, 2021). The ANGPTL4 antibody was validated on the website http://www.ptglab.com/Products/ANGPTL4-Antibody-18374-1-AP.htm#validation.

Animals and other research organisms

Policy information about studies involving animals; ARRIVE guidelines recommended for reporting animal research, and Sex and Gender in Research

Laboratory animals	For FMT experiment, ten-week-old male C57BL/6 mice were raised in conventional environment. For administrating of B. adolescentis, eight-week-old male C57BL/6 mice were raised in conventional environment. Five-week-old male germ-free male C57BL/6J mice were bred and maintained in special plastic isolators. All animals were kept under 12h light-dark cycles at a controlled temperature (23 ± 2 degrees centigrade) and 60–70% humidity, with free access to food and water.
Wild animals	The study did not involve wild animals.
Reporting on sex	As female C57BL/6J mice are reported to be more resistant to the obesogenic effects of HFD (Int J Obes (Lond) 46(10):1749-1758, 2022), male C57BL/6J mice were used in this study.
Field-collected samples	The study did not involve samples collected from field.
Ethics oversight	Animal experimental procedures were approved by the Committee on the Use of Live Animals for Teaching and Research of the University of Hong Kong and Animal Ethics Committee of the Shanghai Sixth People's Hospital Affiliated to Shanghai Jiao Tong University School of Medicine.

Note that full information on the approval of the study protocol must also be provided in the manuscript.

Clinical data

Policy information about clinical studies
All manuscripts should comply with the ICMJE guidelines for publication of clinical research and a completed CONSORT checklist must be included with all submissions.

Clinical trial registration	This trial was registered at Chinese Clinical Trial Registry as ChiCTR-TTRCC 13003333.
Study protocol	The detailed study protocol was deposited in Chinese clinical trial management public platform (Resman) and can be found in Supplementary Information.
Data collection	The CRF was used to collect information during the study. Original files were stored in Shanghai Jiao Tong University Affiliated Sixth People's Hospital. All data were double-entered by researcher staff. After establishing and regarding as correct in blind review, the database was locked, and no alteration can be made. Participants were recruited from July 2013 to October 2016. Data was collected in Shanghai Clinical Center for Diabetes from July 2013 to October 2016.
Outcomes	The primary outcome was change of body weight measured with the body composition analyzer (Tanita, TBF-410) after RS intervention vs. CS intervention, and the secondary outcomes were subcutaneous fat area and visceral fat area assessed using the 3.0 T clinical MRI scanner (Archiva, Philips Medical System), body fat analysis by body composition analyzer, waist circumference by anthropometric measurement, lipid profiles by biochemical assessments, and insulin sensitivity by hyperinsulinemic-euglycemic clamp.

Plants

Seed stocks	*Report on the source of all seed stocks or other plant material used. If applicable, state the seed stock centre and catalogue number. If plant specimens were collected from the field, describe the collection location, date and sampling procedures.*
Novel plant genotypes	*Describe the methods by which all novel plant genotypes were produced. This includes those generated by transgenic approaches, gene editing, chemical/radiation-based mutagenesis and hybridization. For transgenic lines, describe the transformation method, the number of independent lines analyzed and the generation upon which experiments were performed. For gene-edited lines, describe the editor used, the endogenous sequence targeted for editing, the targeting guide RNA sequence (if applicable) and how the editor was applied.*
Authentication	*Describe any authentication procedures for each seed stock used or novel genotype generated. Describe any experiments used to assess the effect of a mutation and, where applicable, how potential secondary effects (e.g. second site T-DNA insertions, mosiacism, off-target gene editing) were examined.*
