# Supplementary figures and images for: Resistant starch intake facilitates weight loss in humans by reshaping the gut microbiota
Source: Nat Metab. 2024 Feb 26;6(3):578–97. doi: 10.1038/s42255-024-00988-y (PMC10963277; doi:10.1038/s42255-024-00988-y)

# Extended Data Figure 8H

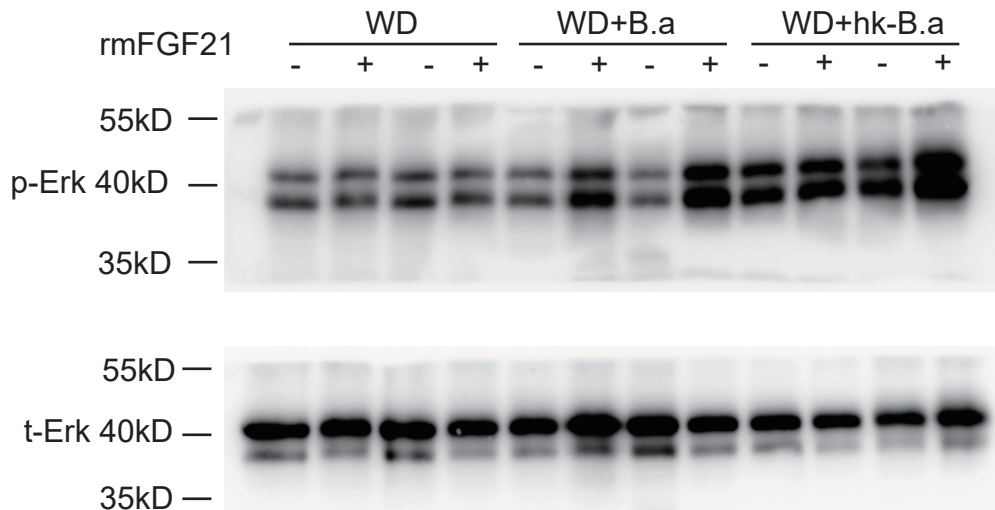

Supplement: Supplementary file 16 — Unprocessed western gels. [file 42255_2024_988_MOESM16_ESM.pdf]
